# Mirabegron treatment reduces myofibroblasts and CXCR2 expression in adipose tissue in obesity

**DOI:** 10.1186/s10020-025-01368-2

**Published:** 2025-10-14

**Authors:** Brian S. Finlin, Hasiyet Memetimin, Philip M. Westgate, Jin Chen, Esther E. Dupont-Versteegden, Philip A. Kern

**Affiliations:** 1 Department of Internal Medicine, Division of Endocrinology, College of Medicine, Lexington, USA; 2 Barnstable Brown Diabetes and Obesity Center, Lexington, USA; 3 College of Public Health, University of Kentucky, Lexington, USA; 4 Department of Medicine, Division of Nephrology, University of Alabama at Birmingham, Birmingham, USA; 5 Department of Rehabilitation Sciences, College of Health Sciences, Center for Muscle Biology, University of Kentucky, Lexington, KY 40536 USA; 6 Division of Endocrinology, CTW 521 University of Kentucky, 900 S. Limestone St, Lexington, KY 40536 USA

**Keywords:** Adipocyte, Adipose tissue physiology, Chemokine receptor, Fibrosis, Gene expression analysis

## Abstract

**Background:**

Treatment with the β3-adrenergic receptor (AR) agonist mirabegron improves insulin sensitivity β, cell function, and glucose tolerance in individuals with obesity, without weight loss or a change in brown adipose tissue (BAT) (Finlin et al, J Clin Invest. 2020 May 1;130(5):2319-2331). Furthermore, mirabegron treatment increased protein expression of beige adipose markers and the number of anti-inflammatory macrophages, and changed the expression of genes involved in fibrosis and tissue remodeling in subcutaneous white adipose tissue (SC WAT).

**Methods:**

We utilized RNA seq and enrichment analysis to identify biological pathways changed by mirabegron treatment in SC WAT thigh biopsies. We verified these changes by immunohistochemistry and performed mechanistic studies in differentiated human adipocytes in vitro.

**Results:**

Mirabegron treatment reduced myofibroblasts, which are fibrotic, and reduced CXCR2, which is involved in inflammation and chemotaxis, in SC WAT. Adipose tissue myofibroblasts were higher with obesity and negatively correlated with β cell function. Mirabegron inhibited TGFβ induction of the adipocyte mesenchymal transition pathway in differentiated adipocytes in vitro. Furthermore, mirabegron treatment reduced expression of *snail*, a transcription factor which promotes the mesenchymal transition pathway, in vitro and in vivo. We also found that mirabegron treatment reduced *CXCR2* expression in SC WAT. CXCR2 was expressed by NK cells and mirabegron treatment reduced CXCR2 expression in NK cells in SC WAT.

**Conclusion:**

Together, these results suggest new mechanisms for improvement of the human SC WAT phenotype by mirabegron treatment to enhance glucose metabolism. These new mechanisms involve the reduction of myofibroblasts as well as the reduction of CXCR2 expression in NK cells.

**Supplementary Information:**

The online version contains supplementary material available at 10.1186/s10020-025-01368-2.

## Introduction

White adipose tissue (WAT) functions to store energy, but is also recognized as an endocrine organ that affects distal organs to influence metabolism and nutrient intake (Rosen and Spiegelman 2014). In obesity, WAT dysfunction occurs resulting in low-grade adipose tissue inflammation, which contributes to insulin resistance and glucose intolerance (Crewe et al. 2017; Sun et al. 2013). The pathway leading to adipose tissue dysfunction is complicated, but is thought to involve hypoxia, fibrosis, and macrophage recruitment, leading to inflammation (Crewe et al. 2017; Sun et al. 2011). In contrast to WAT, brown adipose tissue (BAT) oxidizes lipids to generate heat through uncoupled respiration. A second type of thermogenic adipocyte, called a beige adipocyte, can be induced in WAT in response to cold or various other stimuli (Wu et al. 2012). Both BAT and beige adipose are associated with many beneficial metabolic effects (Cohen and Kajimura 2021), leading to considerable interest in understanding mechanisms to increase and activate thermogenesis in these types of adipose tissue to improve metabolic homeostasis.

The identification of BAT in adult humans (Marken Lichtenbelt et al. 2009; Saito et al. 2009; Virtanen et al. 2009; Cypess et al. 2009), interest in beige adipocyte formation in WAT (Wu et al. 2012; Finlin et al. 2018), and the development of the selective β3-AR agonist mirabegron have led to recent clinical studies in humans. The β3-AR is expressed in both BAT and WAT, and has been thought to be a useful target to improve metabolic homeostasis for decades (Arch 2002, 2011). The initial study of mirabegron demonstrated that treatment of lean, healthy men with a high dose of mirabegron significantly increased BAT activity (Cypess et al. 2015). Subsequently, two studies involving chronic treatment with mirabegron were performed. In one study, older, insulin-resistant research participants with obesity who had little detectable BAT at baseline were treated with mirabegron for 10 to 12 weeks, resulting in improved glucose homeostasis (Finlin et al. 2020). The underlying mechanism for this improvement was a combination of improved insulin sensitivity and β cell function (Finlin et al. 2020). Notably, mirabegron treatment did not increase BAT activity, but stimulated subcutaneous (SC) WAT beiging and lipolysis, and increased type I fibers in muscle (Finlin et al. 2018, 2020). Another recent study found that treatment of young, lean subjects who had BAT at baseline with mirabegron resulted in similar improvements in insulin sensitivity and β cell function, but also increased BAT (Baskin et al. 2018; O’Mara et al. 2020). There were differences between lean and obese subjects in terms of the presence of BAT and its activation, but both studies showed improved insulin sensitivity, β cell function, and glucose homeostasis (Finlin et al. 2020; O’Mara et al. 2020). β cells and muscle do not express the β3-AR, suggesting an indirect mechanism for the improved function involving intra-organ crosstalk.

WAT influences insulin sensitivity and β cell function (Stern et al. 2016), and expresses the β3-AR, suggesting that it mediates the beneficial effects of mirabegron treatment on glucose homeostasis. Here we performed RNA seq on WAT from mirabegron treated subjects to identify potential mechanisms for the improvement in WAT function and identified two novel mechanisms involving myofibroblasts and NK cells.

## Materials and methods

### Human research participants and study design

The research participants and study design have previously been described (Finlin et al. 2020). In brief, all subjects had a mean BMI of 32.4 and were either pre-diabetic or had normal glucose tolerance and at least three features of metabolic syndrome. No subjects had any evidence of chronic inflammatory diseases and were not taking anti-inflammatory or β-blocking drugs. Adipose biopsies from the thigh and abdomen were performed while the research participants were fasting before and after 12 weeks of treatment with mirabegron 50 mg/day. The majority of subjects were female (11 of 13), and we were therefore not able to consider sex as a biological variable. All subjects gave informed consent, and the protocols were approved by the Institutional Review Board at the University of Kentucky. The Clinicaltrials.gov registration identifiers are NCT02596776 and NCT02919176.

### RNA seq

Total RNA was isolated from thigh SC WAT using the RNeasy Mini Kit (#74106, Qiagen) according to the manufacturer’s instructions. RNA integrity was assessed using an Agilent bioanalyzer and RNA was submitted to Novogene for RNA seq analysis. RNA counts were transformed into transcripts per million (TPM). For differential expression analysis, the data were filtered to eliminate very low or non-expressed mRNA using the criteria that half of the fields of data had greater than 1 TPM expression. P-values were determined by a paired, 2-tailed Student’s T-test, and FDR adjustment was done using Benjamini-Hochberg. The RNA seq data are available at the gene expression omnibus (GSE272175). The data were analyzed by gene set enrichment analysis (GSEA), and additionally, a subset of differentially regulated genes was submitted to NIH DAVID and the molecular signatures database for enrichment analysis (Subramanian et al. 2005; Huang da et al. 2009; Sherman et al. 2022).

### mRNA quantification

Total RNA was isolated using the RNeasy Mini Kit (#74106, Qiagen) according to the manufacturer’s instructions. The RNA concentration and quality were measured using Nanodrop (Thermo Fisher). 0.5 ug RNA was used for cDNA synthesis with qScript cDNA Synthesis Kit (#95047-100, Quanta bio). Quantification real-time PCR (qRT-PCR) was performed using PowerUp SYBR green master mix (#2664573, Applied Biosystems). RT-PCR reactions were run on 1-Step RT-PCR program (QuantStudio3, Applied Biosystems) using an equal amount of cDNA from each sample. The amount of mRNA was normalized with the Geomean of six reference genes (ACTA, PPIA, PPIB, TBP, TUBB, UBC9), and the fold change was calculated using the 2-ΔΔCT method. The primer sequences are shown in Table S1.

### Immunohistochemistry

Immunofluorescence double staining was performed to identify myofibroblasts. The concentrations of the primary and secondary antibodies as well as the antigen retrieval conditions are available in Table S2. Briefly, paraffin-embedded SC WAT sections were deparaffinized, subjected to antigen retrieval, treated with 3% hydrogen peroxide for seven minutes to block endogenous peroxidase activity, blocked with 2.5% horse serum, treated with streptavidin/biotin (#SP-2002, Vector Labs, Burlingame, CA), and then incubated with the primary antibodies rabbit anti-vimentin (#ab92547, abcam, Boston MA) and mouse anti-ASMA clone ASM-1 (#CBL171, MilliporeSigma, Rockville, MD) overnight. Sections were rinsed and incubated with the secondary antibodies goat anti-rabbit IgG Alexa Fluor Plus 555 (#A32732, Life Technologies) and goat anti-mouse IgG2a Alexa Fluor 488 (#A21131, Life Technologies). For CXCR2 and NK cell staining, SC WAT sections were blocked in 2.5% horse serum for 2 h, incubated with the primary antibody rabbit anti-CD158d/KIR2DL4 (#PA5-115470, Life Technologies) overnight, washed, and then incubated with the secondary antibody biotinylated goat anti-rabbit antibody (Jackson ImmunoResearch, catalog 111-065-003), followed with streptavidin-HRP (#S911, Life Technologies). Slides were then rinsed and incubated with AlexaFluor 594 tyramide reagent (Invitrogen, catalog B40957). Sections were blocked with 5% horse serum for one hour, incubated overnight with the second primary antibody mouse IgG2a anti-human CXCR2/IL-8 RB antibody (#MAB331, R&D system), rinsed, and incubated with biotinylated goat anti-mouse IgG2a antibody (Jackson ImmunoResearch, catalog 115-065-206), along with Streptavidin-HRP (#S911, Life Technologies), rinsed and incubated with AlexaFluor 488 tyramide reagent (Invitrogen, catalog B40953). Coverslips and VECTASHIELD antifade mounting medium with DAPI (#H-1800, Vector Lab, Newark, CA) were applied to the slides. Images were captured with a Zeiss AxioImager MI upright fluorescent microscope (Zeiss, Gottingen, Germany), and analysis was performed using Zen software (Zeiss). Approximately 10 fields (20X) were counted per subject. Myofibroblasts were counted as DAPI (blue), vimentin (red), and ASMA (green) co-localized cells; fibrotic areas and vascular areas were excluded. Myofibroblast numbers were normalized to the number of adipocytes. NK cells co-stained with CXCR2 were identified as DAPI (blue), CXCR2 (green), CD158d (red) colocalized cells and normalized to adipocyte number.

For PDGFRA and ASMA, or PDGFRA and vimentin co-staining, after all blocking steps, the sections were incubated overnight with goat anti-PDGFRA (R&D system, AF-307-NA), washed, and incubated with biotinylated donkey anti-goat IgG (Jackson ImmunoResearch, catalog #705-065-147) secondary antibody, followed by streptavidin-HRP. The sections were then rinsed and incubated with AlexaFluor 594 tyramide reagent. After blocking with 5% horse serum for 1 h, the sections were incubated overnight with a second primary antibody, mouse IgG2a anti-ASMA antibody or rabbit anti-vimentin antibody. The sections were rinsed and incubated with biotinylated donkey anti-mouse IgG (Jackson ImmunoResearch, 705-065-150) or biotinylated donkey anti-rabbit IgG (Jackson ImmunoResearch, 711-065-152) antibody, followed by Streptavidin-HRP, and then treated with AlexaFluor 488 tyramide reagent. For SMA and perilipin, after antigen retrieval, hydrogen peroxide and streptavidin/biotin blocking, sections were blocked with 2.5% horse serum. The sections were incubated overnight with the rabbit anti-perilipin (Cell Signaling, 9349) and mouse anti-SMA (Sigma, CBL171), washed, and incubated goat-anti rabbit Alex 555 (Life Technologies A32732) and goat anti-mouse IgG2a Alex 488 (Life Technologies A21131). For CD31 and ASMA, or CD31 and vimentin co-staining, the sections were incubated overnight with goat anti-CD31 (R&D system, AF3628) and mouse anti-SMA (Sigma, CBL171), or goat anti-CD31 and rabbit-anti vimentin (Abcam, AB92547), washed, and incubated with donkey anti-goat Alex 647 (Life Technologies A32849) and donkey anti mouse Alex 488 (Life Technologies A21202), or donkey anti-goat Alex 647 (Life Technologies A32849) and donkey anti-rabbit Alex 555 (Life Technologies A31672). For CD68 and ASMA, or CD68 and vimentin co-staining, after all blocking steps, the sections were incubated overnight with the mouse anti-CD68 (Abcam, AB955) or rabbit anti-CD68 (Cell signaling #76437), washed, and incubated with the biotinylated goat anti-mouse IgG1 (Jackson ImmunoResearch, 115-065-205) or biotinylated goat anti-rabbit IgG (Jackson ImmunoResearch,111-065-003) secondary antibody, followed by streptavidin-HRP. The sections were then rinsed and incubated with AlexaFluor 594 tyramide reagent (Invitrogen, B40957). After blocking with 5% horse serum for 1 h, the sections were incubated overnight with the second primary antibody, mouse IgG2a anti-ASMA antibody or rabbit anti-vimentin. The sections were rinsed, and incubated with fluorescence-conjugated second antibodies, goat anti-mouse Ig2a Alex 488 or goat anti-rabbit Alex 488. Coverslips and VECTASHIELD antifade mounting medium with DAPI (#H-1800, Vector Lab, Newark, CA) were applied to the slides. For all antibody reactions, negative controls were used in which no primary antibody was applied, and no staining was observed. Images were captured with a Zeiss AxioImager MI upright fluorescent microscope (Zeiss, Gottingen, Germany), and analysis was performed using Zen software (Zeiss).

### Differentiated human adipocyte culture experiments

Adult-derived human adipocyte stem cells (ADHASCS) were cultured in 10% fetal bovine serum (FBS) in 1:1 DMEM/F10 (Dulbecco’s modified eagle medium (Gibco, Invitrogen 11885-084)/Ham’s F-10 with L-glutamine (Corning, Invitrogen 10-070-CV) until they reached 100% confluence. To induce to differentiation, the cells were cultured in induction medium (3%FBS in DMEM/F10 containing 100 nM insulin (Novolin R, Novo Nordisk Inc, Plainsboro, NJ), 1.0 mM dexamethasone (D2915, Sigma-Aldrich), 0.25 mM 3-isobutyl-1-methylxanthene (IBMX, I5879, Sigma-Aldrich), 0.033 mM biotin (B4639, Sigma-Aldrich), 0.017 mM pantothenate (P5155, Sigma-Aldrich), and 1.0 mM rosiglitazone (71740, Cayman) for 3 days. Afterward, the medium was replaced with maintenance medium (induction medium without IBMX and rosiglitazone) for 7 days, with fresh medium added every other day. Following 10 days of differentiation, the cells were pre-treated with 100 nM mirabegron in 3% FBS DMEM/F10 for 6 h. The medium was then replaced with medium containing 5 ng/mL TGFβ2 and 100 nM mirabegron in 3% FBS in DMEM/F10 for a total of 72 h with a medium change after 48 h. This experiment was also performed with 10 µM isoproterenol (Sigma, I5627) or 2 µM forskolin (Sigma, F3917) instead of mirabegron. The cells were collected separately for RNA or protein analysis (see below).

### Immunoblot analysis of ASMA

Differentiated ADHASCs were treated with or without TGFβ2 (5 ng/mL) and mirabegron (100 nM), or isoproterenol (10 µM), or forskolin (2 µM) as indicated for 72 h as described above. The cells were lysed in RIPA buffer (50 mM Tris-HCl, pH 7.4, 150 mM NaCl, 1% Triton X-100, 0.1% SDS, 0.5% sodium deoxycholate, 2 mM EDTA, 50 mM sodium fluoride, and Halt Protease & Phosphatase Inhibitor Cocktail (#1861281, Thermo Fisher). The protein concentration was determined using the Coomassie Plus Protein Assay Reagent (#1856210, Thermo Fisher). 5 µg of protein was loaded onto 12% precast polyacrylamide gels (#4561046, Bio-Rad) and transferred to a nitrocellulose membrane. The membrane was stained with Ponceau S stain and blocked with 5% BSA in TBS-T (20 mM Tris, 137 mM NaCl, pH 7.6 with 0.1% Tween 20) at room temperature for 1 h and incubated overnight at 4 °C with mouse anti αSMA (1:1,000; Sigma, CBL171). The next day, the membrane was washed in TBST and incubated with goat anti-mouse IgG2a HRP-conjugated antibody (1:10,000; Invitrogen, #M32207) for 1 h at room temperature. Blots were developed using Clarity Western ECL Substrate (Bio-Rad, #170–5060), and images were captured using the ChemiDoc Imaging System (Bio-Rad, USA). ASMA was quantified and then divided by total protein determined from quantification of the Ponceau S stain to determine the ratio. The average of the controls was determined, and the data including each of the controls were normalized to this.

### Chamber slide experiments

Human ADHASCs were cultured in 8-well Lab-Tek chamber slides (Thermo Fisher, #177445), differentiated, and treated with mirabegron and TGFβ2 as described above. After treatment, the cells were incubated with fixation buffer (BioLegend, 420801) for 20 min in the dark at room temperature, washed with PBS, blocked with staining buffer (#554656, BD Pharmingen) for 1 h at RT, and stained overnight at 4 °C with smooth muscle anti-actin antibody, clone ASM-1 (#CBL171, MilliporeSigma, Rockville, MD) and anti-perilipin-1 antibody (#9349, Cell signaling). The cells were rinsed and then incubated with goat anti-mouse IgG2a Alexa Fluor 488 (#A21131, Life Technologies) and goat anti-rabbit IgG Alexa Fluor Plus 555 (#A32732, Life Technologies) for 1 h at room temperature. The cells were then washed and mounted with VECTASHIELD anti-fade mounting medium containing DAPI (#H-1800, Vector Labs, Newark, CA). Images were captured using a Zeiss AxioImager MI upright fluorescent microscope (Zeiss, Göttingen, Germany). ASMA expression was quantified using Zen software (Zeiss) by setting a threshold for ASMA fluorescence. The ASMA threshold was normalized to the number of DAPI-positive cells in each 20x magnification field.

### Statistics

For analysis of ASMA expression in ADHASC cells by immunohistochemistry or immunoblotting, we used a linear model with the Satterthwaite method to account for unequal variances. Analyses were conducted using the MIXED procedure in SAS v9.4 (SAS Institute, Cary, NC, USA). Otherwise, paired, 2-tailed Student’s t tests, 2-way ANOVA, and linear regression analyses were conducted in Graphpad Prism version 10.0.2 as indicated. Principal component analysis of the RNA seq data was performed in R Studio.

## Results

### SC WAT mRNA expression analysis

We performed RNA seq on thigh adipose tissue biopsies obtained before and after mirabegron treatment of obese research participants (Finlin et al. 2020). Principal component analysis and a volcano plot are shown in Figure S1. The principal component analysis suggested only a modest effect of mirabegron treatment on adipose tissue gene expression. Nevertheless, we did find 173 genes with false discovery rate (FDR) adjusted P-values less than 0.05 and a 1.5-fold change in expression (Table S3). We performed GSEA to gain insight into the biological processes affected by mirabegron treatment (Subramanian et al. 2005). As shown in Table 1, four gene sets (Liberzon et al. 2011) were enriched in the baseline (pre mirabegron treatment) controls (*P* < 0.001; FDR adjusted *P* < 0.05); no other gene sets were detected with FDR *P* < 0.05 in the baseline or post mirabegron treatment biopsies. The enrichment plots are shown in Figure S2, the biological processes in Table S4, and the lists of genes in each of the four gene sets are available in Tables S5 through S8. Three of the differentially expressed gene sets include breast cancer. Epithelial to mesenchymal stem cell transition (EMT) is a hallmark of breast cancer and mesenchymal transition is utilized in trans-differentiation of various cell types into myofibroblasts. SC WAT contains myofibroblasts and their role in adipose tissue has recently been explored (Bourlier et al. 2012; Divoux et al. 2010). However, SC WAT is not known to contain an epithelial cell population, so we investigated the expression and regulation of genes in the classical EMT pathway (Lamouille et al. 2014), and found that they were not changed (Table S9). Notably, the expression of keratins 8 and 18, E-cadherin, and N-Cadherin were quite low, and not changed by mirabegron treatment. These findings suggest that the classical pathway of EMT, in which epithelial cells undergo mesenchymal transition, is not involved in the transcriptional response to mirabegron in adipose tissue. Nevertheless, the genes identified by GSEA as being enriched in the baseline biopsies (Tables S5 through S8) are involved in mesenchymal transition, suggesting that mirabegron treatment possibly affected an alternative pathway, in which cells other than epithelial cells are transformed into myofibroblasts in SC WAT. Indeed, recent studies indicate that other cells in adipose tissue including adipocytes are capable of differentiating into myofibroblasts by mesenchymal transition (Zhang et al. 2019; Marangoni et al. 2015). Therefore, we proceeded to determine whether mirabegron treatment reduced myofibroblasts in adipose tissue.

**Table 1:** Enrichment of curated gene sets in thigh subcutaneous adipose Tissue^a^

Gene sets enriched in baseline (pre mirabegron treatment) biopsies					
NAME	SIZE	ES	NES	NOM p-val	FDR q-val
LIM_MAMMARY_LUMINAL_PROGENITOR_UP	54	0.63	1.94	< 0.001	0.026
SMID_BREAST_CANCER_ERBB2_UP	137	0.60	1.95	< 0.001	0.033
ROY_WOUND_BLOOD_VESSEL_DN	21	0.76	1.91	< 0.001	0.043
LIEN_BREAST_CARCINOMA_METAPLASTIC_VS_DUCTAL_DN	100	0.57	1.92	< 0.001	0.043

α-smooth muscle actin (ASMA) is used to identify myofibroblasts, but is present in other cells such as pericytes and smooth muscle cells, which are localized in blood vessels (Younesi et al. 2024). Therefore, to identify myofibroblasts, we performed immunohistochemical co-staining with vimentin and ASMA antibodies and excluded cells in vessel structures. Hematoxylin and eosin staining of thigh SC WAT did not reveal changes to the adipose tissue structure (Figure S3), consistent with our previous characterization of abdominal SC WAT (Finlin et al. 2021). We tested the specificity of the vimentin and ASMA antibodies as follows. First, we confirmed that the vimentin antibody identified fibroblasts using PDGFRA, a fibroblast marker, and observed that vimentin and PDGFRA displayed considerable co-staining (Figure S4A). We also observed that ASMA is present in PDGFRA positive cells (Figure S4B). These two results indicate that the vimentin antibody identifies fibroblasts in SC WAT, some of which are myofibroblasts. Next, we determined whether co-staining with vimentin and ASMA would exclude adipocytes, endothelial cells, and macrophages. ASMA did not co-stain with perilipin in unilocular adipocytes (Figure S5A) or in smaller cells in between adipocytes (Figure S5B). ASMA co-stained with some CD31 positive cells, which is marker of endothelial cells (Figure S6A); however, vimentin did not exhibit any co-staining with CD31 (Figure S6B). ASMA also co-stained some cells positive for the macrophage marker CD68 (Figure S7A), but there was little co-staining with vimentin and CD68 (Figure S7B), indicating that macrophages would mostly be excluded by our ASMA and vimentin co-staining. Together these results indicate that vimentin and ASMA co-staining identifies myofibroblasts in SC WAT, but it is possible that some macrophages expressing ASMA would be detected.

**Fig. 1:**
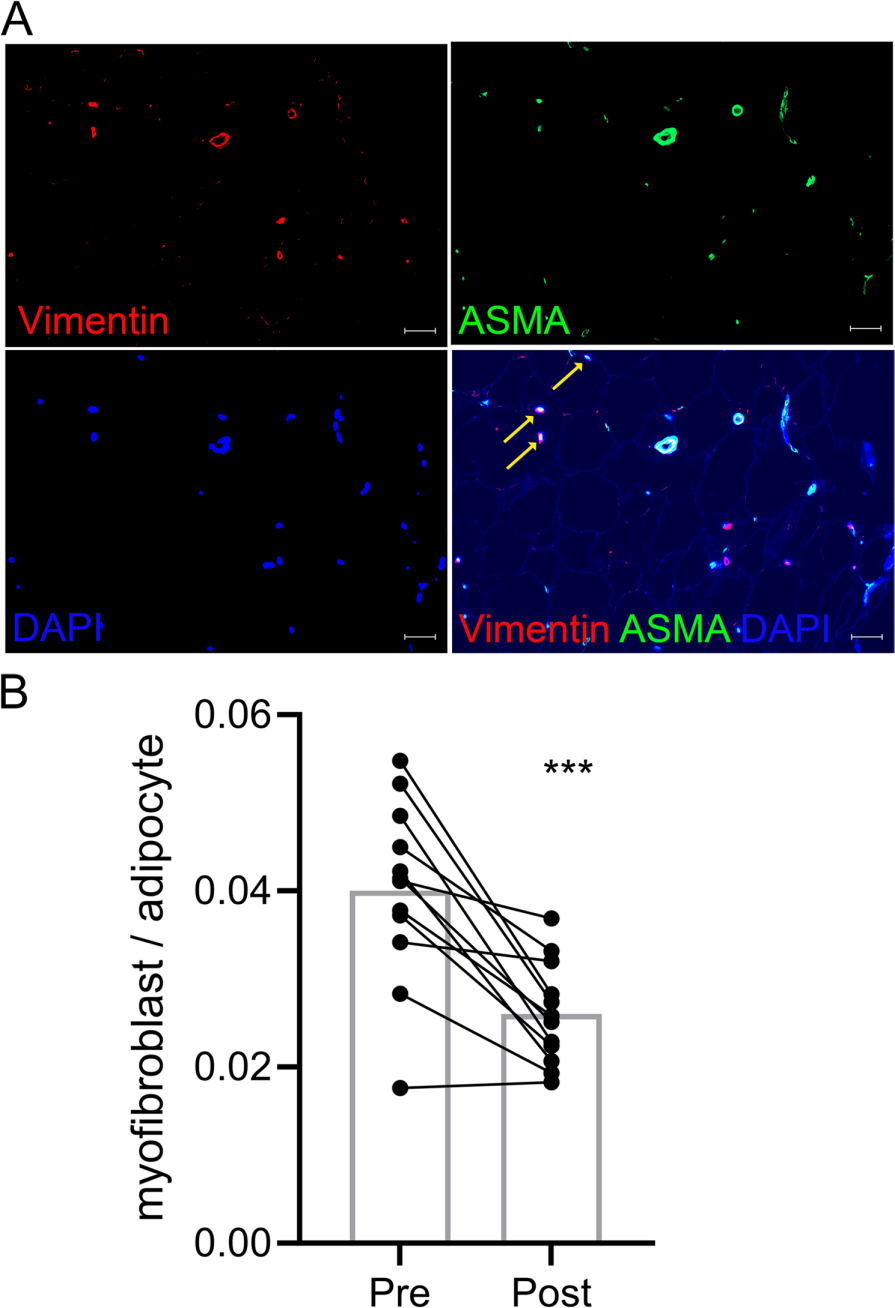
Mirabegron treatment decreases adipose tissue myofibroblasts. **A** Identification of myofibroblasts in obese adipose tissue by immunohistochemistry. Details about the antibody concentrations used are found in Table S2. The results of vimentin, ASMA, and DAPI immunohistochemical staining are shown in each individual channel followed by the merged image (scale bar: 50 μm). Yellow arrows point to co-stained myofibroblasts; cells in blood vessels are positive for vimentin and ASMA, but are not counted. DAPI channel auto fluorescence shows the outline of the adipocytes in the merged image. **B** Quantification of myofibroblasts in thigh SC WAT before and after mirabegron treatment. Data represent means (*n* = 12) and were analyzed by a paired, 2-tailed Student’s t test; ***P < 0.001

As shown in Fig. 1A, myofibroblasts were usually identified as single cells between adipocytes, consistent with a previous study of SC WAT (Divoux et al. 2010). Mirabegron treatment significantly reduced myofibroblast abundance (myofibroblast per adipocyte) by approximately 1.5-fold in thigh SC WAT (Fig. 1B; *P* < 0.001) as well as in abdominal SC WAT (Figure S8). Mirabegron treatment did not cause weight loss (Finlin et al. 2020) or change adipocyte size (Finlin et al. 2021), making it unlikely that normalization to adipocyte number influenced this finding. In support of this, mirabegron treatment significantly reduced myofibroblast abundance using the myofibroblast normalized to area ratio (Figure S9).

**Fig. 2:**
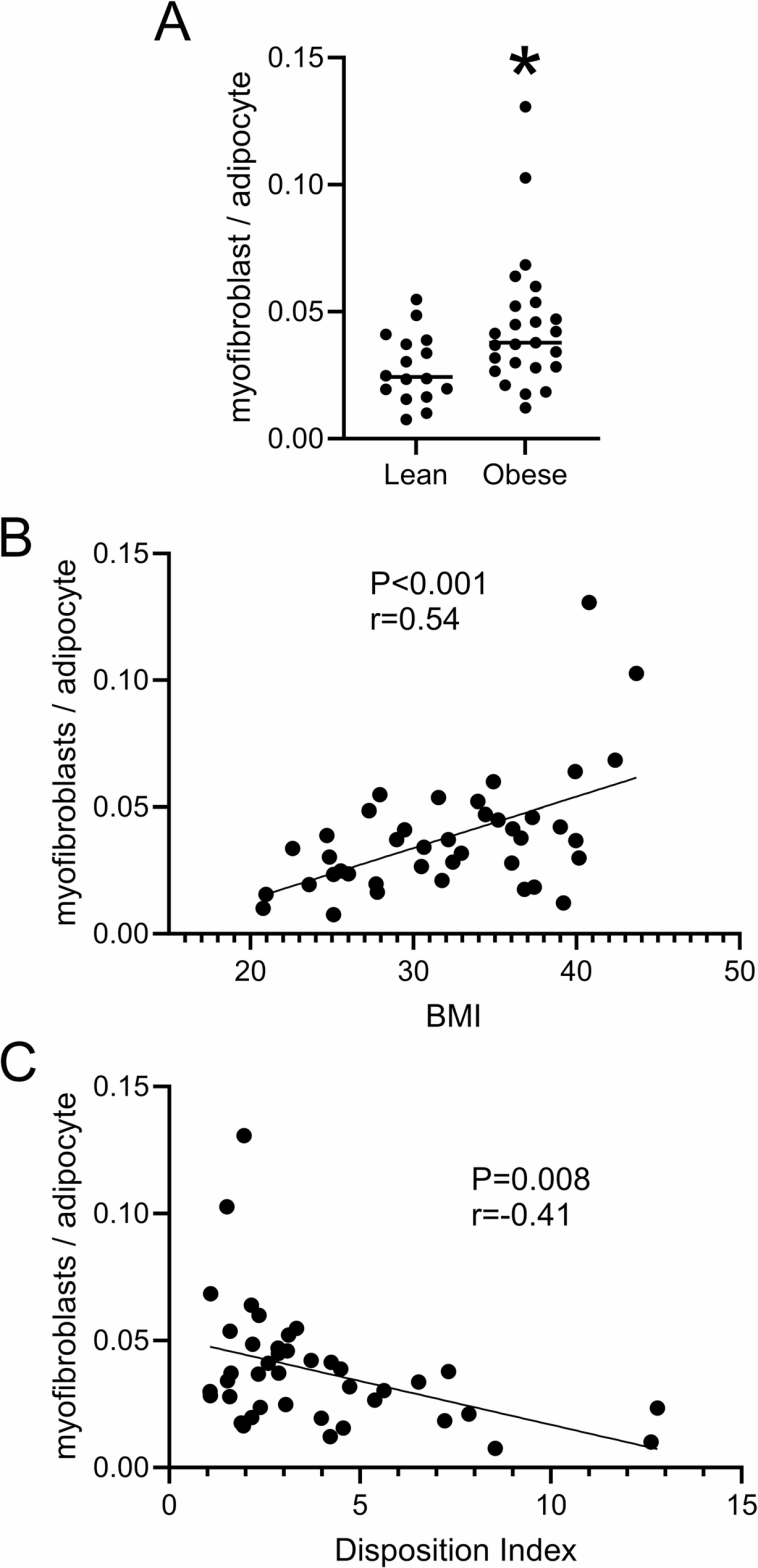
Adipose tissue myofibroblasts are higher with obesity and are inversely correlated with β cell function. **A** Research participants were grouped into lean and obese groups using a BMI cutoff of 30. Myofibroblast density was determined as in Fig. 1. Data represent means (*n* = 16 lean; *n* = 25 obese) and were analyzed by a paired, 2-tailed Student’s t test; *P < 0.05. (**B**) and (**C**) myofibroblast density was correlated to BMI and the disposition index. Data were analyzed by linear regression analysis (*n* = 41); Pearson correlation coefficients and P values are indicated

Myofibroblasts have not been extensively studied in WAT in relation to glucose homeostasis, thus the significance of the reduction in myofibroblasts by mirabegron treatment is not clear. We therefore measured adipose myofibroblasts in thigh SC WAT biopsies (baseline) from additional subjects previously characterized that had a wide range of BMI, insulin sensitivity, and β cell function (Finlin et al. 2018, 2020, 2021). We first divided these into lean (BMI < 30) and obese (BMI > 30) groups. As shown in Table 2, these research participants had significant differences in BMI, insulin sensitivity (Matsuda index), insulin secretion adjusted for insulin sensitivity (disposition index), and baseline glucose as expected. The density of myofibroblasts was higher in the obese group (Fig. 2A), and linear regression analysis indicated that adipose myofibroblasts positively correlated with BMI (Fig. 2B; *r* = 0.54; *P* < 0.001). Next, we performed linear regression analysis of myofibroblast density and insulin sensitivity and the disposition index, a measure of β cell function. We found that myofibroblast density is negatively correlated with the disposition index (Fig. 2C; *r*=−0.41; *P* < 0.01), suggesting a role for myofibroblasts in adipose tissue dysfunction and its negative impact on glucose homeostasis. The relationship between adipose tissue myofibroblast density and insulin sensitivity was negatively correlated but not statistically significant. We previously observed that mirabegron treatment improved the disposition index (Finlin et al. 2020); the reduction in adipose tissue myofibroblasts by mirabegron treatment could thus be an important part of the mechanism by which mirabegron treatment improved β cell function. Finally, we attempted to examine myofibroblasts in muscle because of the numerous positive effects of mirabegron treatment on muscle in obese subjects (Finlin et al. 2020). We identified fibroblasts (vimentin-stained cells) between myofibers, but did not identify myofibroblasts in the baseline muscle biopsies (Figure S10); thus, the effect of mirabegron on myofibroblasts is tissue specific.

**Table 2:** Baseline characteristics of adipose myofibroblast density study subjects

Study^a^	Lean Subjects	Obese Subjects	*P* ^b^
Number (Gender M/F)	16 (3/13)	25 (4/21)	
Age	50.6 ± 4.9	49.7 ± 1.5	0.79
BMI	25.5 ± 0.8	36.2 ± 0.7	< 0.0001
S_I_ (Matsuda)	4.6 ± 0.8	2.5 ± 0.3	< 0.01
Insulinogenic Index	1.2 ± 0.2	1.5 ± 0.2	0.34
Disposition Index	5.0 ± 1.1	3.1 ± 0.4	< 0.05
Fasting Glucose	91 ± 3	98 ± 1.5	< 0.05

**Fig. 3:**
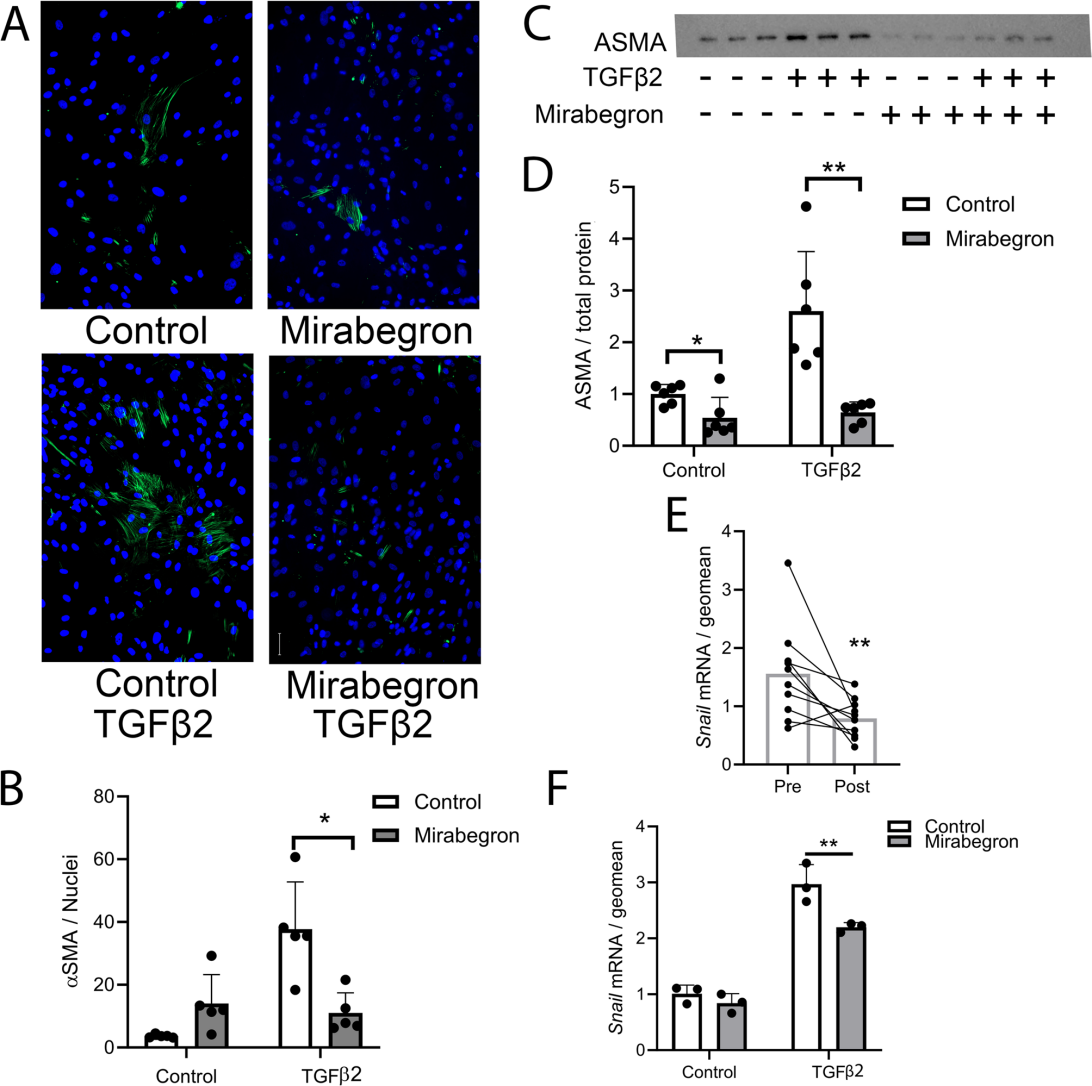
Mirabegron treatment blunts TGFβ induction of ASMA in cultured adipocytes and reduces snail mRNA expression in adipose tissue and in vitro. A - D) Differentiated human adipocytes were treated with or without mirabegron (100 nM) for 6 h. Cells were then treated with or without TGFβ2 (5 ng/mL) in the absence or presence of mirabegron (100 nM) as indicated. ASMA was detected by immunohistochemistry, quantified, and normalized to the number of nuclei (scale bar 50 μm); see Table S2 for details about the antibody concentrations. B) Data represent means ± SD (*n* = 5) and were analyzed by a linear model described in methods; interaction: *P* < 0.05; *P < 0.05. C and D) ASMA was detected by immunoblotting. Data represent means ± SD (*n* = 6) and were analyzed as in B; interaction: *P* < 0.05; *P < 0.05; **P < 0.01. E) Snail mRNA expression was measured in human thigh SC WAT before and after mirabegron treatment. Data represent means (*n* = 10) and were analyzed by a paired, 2-tailed Student’s t test; **P < 0.01. F) Snail mRNA expression was measured in differentiated adipocytes treated with mirabegron and TGFβ2 as described in panel A. Data represent means ± SD (*n* = 3) and were analyzed by two-way ANOVA; *P < 0.05; **P < 0.01

A direct mechanism for the reduction of myofibroblasts in SC WAT by mirabegron would be inhibition of the adipocyte mesenchymal transition pathway. Adipocyte mesenchymal transition can be induced by TGFβ in vitro and occurs in vivo in mice (Zhang et al. 2019; Marangoni et al. 2015; Zhu et al. 2022); cAMP signaling, which would occur through β-adrenergic receptor stimulation, has been shown to inhibit TGFβ signaling in some circumstances (Schiller et al. 2010). Therefore, we tested the ability of mirabegron to inhibit TGFβ induction of ASMA in differentiated human adipocytes. We established an in vitro assay using differentiated human adipocytes. As shown in Figure S11A, TGFβ induced ASMA expression and perilipin was not detected in ASMA positive cells, consistent with a previous study (Marangoni et al. 2015). As a positive control, we determined whether isoproterenol, which activates all βARS, or forskolin, which elevates cAMP directly through adenylyl cyclase, inhibit ASMA induction by TGFβ. As shown in Figure S11B, both compounds significantly inhibited the TGFβ stimulation of ASMA protein (full uncropped blots are available in Figure S12). Next, we tested whether mirabegron blunted the induction of ASMA by TGFβ, and found that it did (Fig. 3A and B; *P* < 0.05). This was confirmed by immunoblot analysis (Fig. 3C and D; *P* < 0.01; full uncropped blots are available in Figure S13). Examination of Table S3 for genes known to regulate mesenchymal transition revealed that the mRNA expression of the transcription factor *snail* (Wang et al. 2013), was repressed by mirabegron treatment. We examined*snail* expression in thigh SC WAT by RT-PCR before and after mirabegron treatment, and confirmed that it was decreased by 2-fold in vivo (Fig. 3E; *P* < 0.01; *n* = 10). Next, we examined *snail* expression in differentiated human adipocytes treated with TGFβ and mirabegron as described above. TGFβ increased *snail* expression and this increase was significantly inhibited by mirabegron treatment (Fig. 3F; *P* < 0.01). Together, the results of this in vitro study suggest that mirabegron inhibits the adipocyte mesenchymal transition pathway, identifying a possible mechanism for the reduction of myofibroblasts in vivo. As mentioned above, we did not identify markers of the classical EMT pathway when we performed differential gene expression analysis of SC WAT. The genes identified in Tables S5 to S8 may be involved an alternative pathway of mesenchymal transition to generate myofibroblasts in adipose tissue from a cell other than an epithelial cell such as an adipocyte.

### CXCR2 expression analysis

**Fig. 4:**
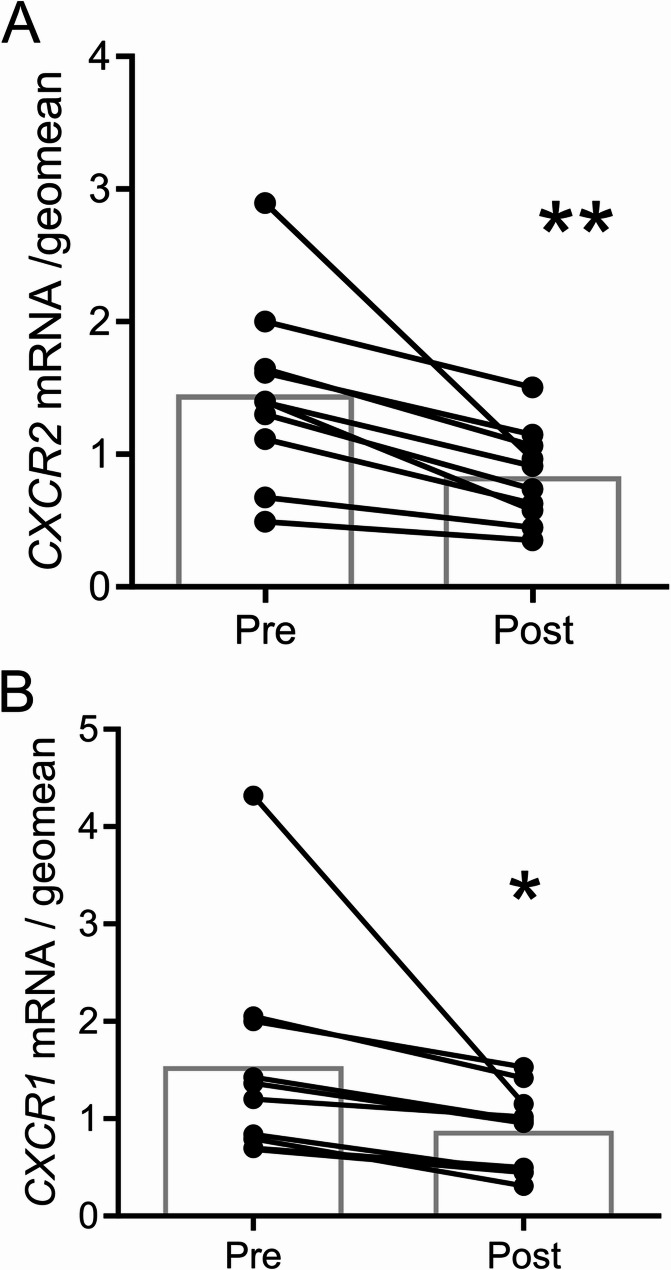
Mirabegron treatment reduces CXCR2 and CXCR1 mRNA expression in thigh SC WAT. (A) and (B) CXCR2 and CXCR1 mRNA expression was determined by qRT PCR. Primer sequences are available in Table S1. Data represent means (*n* = 10) and were analyzed by a paired, 2-tailed Student’s t test; **P < 0.01; *P < 0.05

**Fig. 5:**
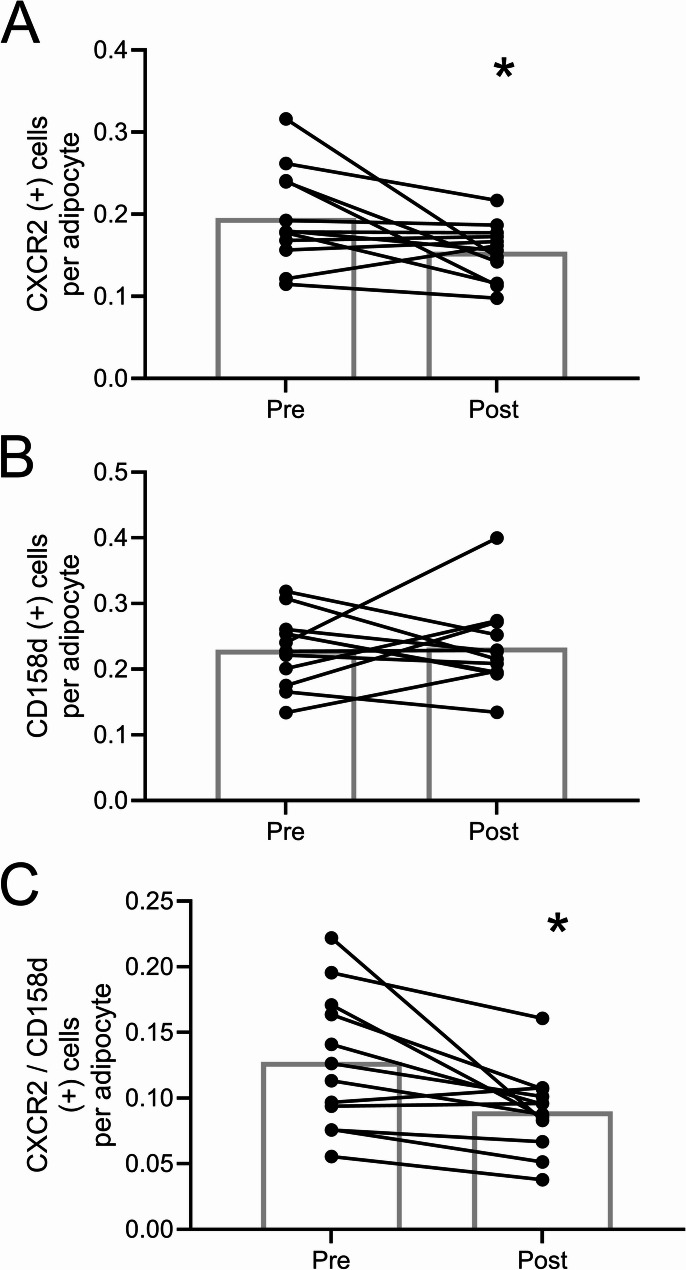
Mirabegron treatment reduces CXCR2 expression in adipose tissue NK cells. A-C) Quantification of CXCR2 positive (+) cells, NK cells (CD158d), and CXCR2 co-stained NK cells (CD158d) by immunohistochemistry. Details about the antibody concentrations used are found in Table S2. Data represent means (*n* = 12) and were analyzed by a paired, 2-tailed Student’s t test; *P < 0.05

Searches of NIH DAVID and the molecular signatures databases with enriched genes identified pathways involving chemokine signaling (Table 3). CXCR2 and CXCR1 are receptors for IL8 and chemokines, and are involved in the chemotaxis of immune cells. CXCR2 is one of the most significantly down regulated genes by mirabegron treatment identified by RNA seq, and CXCR1 was also down-regulated (Table S2). This was verified by RT-PCR (Fig. 4), and we pursued the function of the most significantly down-regulated gene (1.7-fold), *CXCR2*. We queried the human protein atlas data base of single cell seq in adipose tissue (Karlsson et al. 2021) and found that*CXCR2* is highly expressed in NK cells. CXCR2 showed significant co-staining with the NK cell marker CD158d (Faure and Long 2002), the NK marker utilized by the human protein atlas (Figure S14). Mirabegron treatment reduced the number of CXCR2 positive cells in adipose tissue (Fig. 5A; *P* < 0.05); however, it did not reduce the number of NK cells assessed by CD158d staining (Fig. 5B). Mirabegron treatment reduced the expression of CXCR2 on NK cells by 1.4-fold (Fig. 5C; *P* < 0.05), suggesting that mirabegron treatment altered the phenotype of NK cells. Similar results were obtained when we normalized staining to area (Figure S15).

**Table 3:** Molecular signatures gene ontology and NIH DAVID database search results

Gene set name	*P*-Value	FDR adjusted q-value
HP_SEVERE_INFECTION	2.28E-07	3.65E-03
GOCC_SIDE_OF_MEMBRANE	6.41E-07	5.13E-03
GOBP_CELLULAR_DEFENSE_RESPONSE	2.35E-06	1.25E-02
GOMF_C_C_CHEMOKINE_BINDING	1.07E-05	3.12E-02
HP_ANEMIA_DUE_TO_REDUCED_LIFE_SPAN_OF_RED_CELLS	1.16E-05	3.12E-02
GOBP_DENDRITIC_CELL_CHEMOTAXIS	1.37E-05	3.12E-02
GOMF_G_PROTEIN_COUPLED_CHEMOATTRACTANT_RECEPTOR_ACTIVITY	1.37E-05	3.12E-02
GOBP_DENDRITIC_CELL_MIGRATION	2.84E-05	4.53E-02
GOMF_CHEMOKINE_BINDING	2.84E-05	4.53E-02
HP_SEVERE_INFECTION	2.28E-07	3.65E-03

Database: NIH David		
Term	*P*-Value	Benjamini
C-C chemokine binding	5.65E-04	0.027956
C-C chemokine receptor activity	5.65E-04	0.027956
cellular defense response	1.17E-04	0.032413
chemokine-mediated signaling pathway	2.54E-04	0.032413
dendritic cell chemotaxis	3.58E-04	0.032413
neutrophil chemotaxis	4.20E-04	0.032413

### Summary

**Fig. 6:**
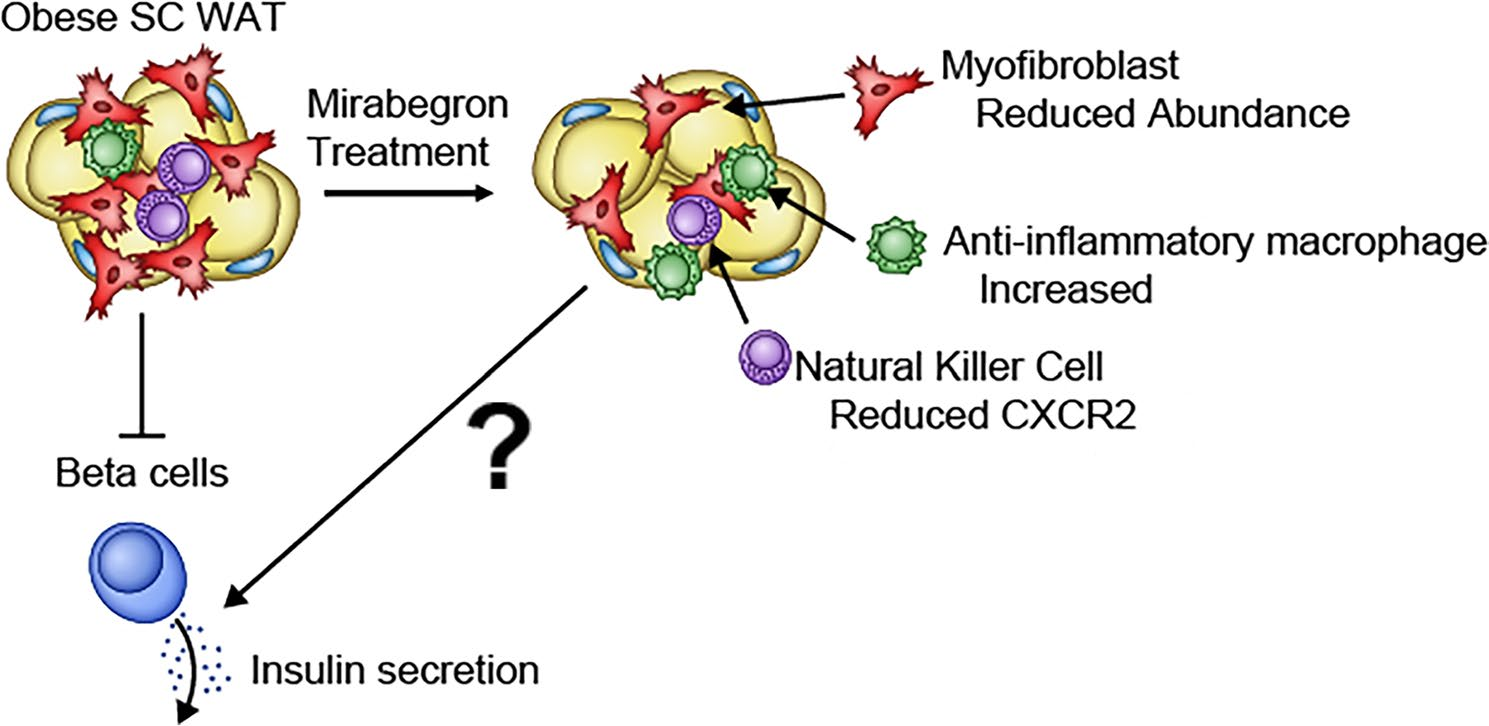
Model for mechanism by which mirabegron treatment improves glucose homeostasis in human research participants with obesity. Mirabegron treatment reduces myofibroblast abundance and changes the phenotype of NK cells to reduce SC WAT dysfunction. This in turn improves b-cell function by a mechanism that remains to be determined

In summary, mirabegron treatment reduced the abundance of myofibroblasts in SC WAT and inhibited the adipocyte mesenchymal transition pathway in vitro. Myofibroblasts increase in adipose tissue with obesity and are negatively correlated with β cell function. Mirabegron treatment reduced CXCR2 expression on NK cells. Together these findings provide new insights into the mechanisms by which mirabegron treatment improves glucose tolerance in humans (Fig. 6).

## Discussion

This study utilized an unbiased approach to identify the effect of mirabegron treatment on adipose tissue gene expression. Enrichment analysis suggested that mirabegron inhibited -mesenchymal transition in adipose tissue and reduced chemotactic and inflammation pathways mediated by CXCR2. Mesenchymal transition is a critical process in the trans-differentiation of various cell types into myofibroblasts. We verified that adipose tissue myofibroblasts are reduced by mirabegron treatment and demonstrated that mirabegron inhibits the adipocyte mesenchymal transition pathway in vitro. Mirabegron treatment reduced CXCR2 expression on adipose tissue NK cells; however, the abundance of NK cells was not changed. Thus, mirabegron treatment affects two cell types that have recently been implicated in the development of adipose tissue fibrosis and inflammation: myofibroblasts and NK cells. Fibrosis and inflammation are both important features of adipose tissue dysfunction in obesity in mice and humans (Crewe et al. 2017; Sun et al. 2011; Spencer et al. 2010). The reduction in myofibroblasts and change in the NK cell phenotype in adipose tissue are likely mechanisms contributing to improvement in glucose homeostasis by mirabegron treatment (Finlin et al. 2020; O’Mara et al. 2020).

Myofibroblasts have been implicated in development of adipose tissue fibrosis (recently reviewed (Marcelin et al. 2019), but have not been extensively studied in human SC WAT, where fibrosis has been demonstrated to increase with obesity (Spencer et al. 2010). Divoux et al. found that myofibroblasts are located in fibrotic bundles of omental fat, and Bourlier et al. found that*snail* mRNA is increased in adipose tissue with obesity (Bourlier et al. 2012; Divoux et al. 2010). These studies are thus consistent with our observation of increased myofibroblast abundance in SC WAT with obesity. The development of adipose dysfunction is a complicated process, and adipose tissue fibrosis has been identified as a critical part of the process (reviewed in (Crewe et al. 2017; Sun et al. 2013; Marcelin et al. 2019). Myofibroblasts may be involved in tissue remodeling in WAT that occurs with obesity. However, myofibroblasts contribute to pathological fibrosis in a number of physiological settings (Younesi et al. 2024), and we found that adipose tissue myofibroblast abundance was negatively correlated with β cell function. This suggests that SC WAT myofibroblast density contributes to adipose tissue dysfunction and has a negative impact on WAT to β cell cross talk, consistent with a study linking myofibroblast precursors with obesity and metabolic dysfunction in mice (Marcelin et al. 2017). It is well established that adipose tissue communicates with β cells via secreted factors such as adiponectin, leptin, and other mechanisms (Stern et al. 2016). However, mirabegron treatment did not affect the plasma levels of adiponectin, leptin, or inflammatory cytokines (Finlin et al. 2020). Therefore, identifying the mechanism by which mirabegron improves β cell function and whether reduction of myofibroblasts in SC WAT is part of the mechanism is an important future goal. The results of this study provide new insight into the role that myofibroblasts play in the development of adipose dysfunction and its effect on peripheral tissues.

This study identifies the β3-AR agonist mirabegron as a pharmacologic agent that reduces SC WAT myofibroblasts. Mirabegron inhibited the adipocyte mesenchymal transition pathway in vitro. The utilization of this pathway to regulate SC WAT myofibroblast density in vivo has not been determined. However, adipocyte mesenchymal transition occurs in adipose depots besides SC WAT and contributes to other diseases. Lineage tracing studies have demonstrated that adipocyte mesenchymal transition in dermal adipose tissue promotes scleroderma (Zhang et al. 2019; Marangoni et al. 2015) and in breast adipose tissue promotes breast cancer progression (Zhu et al. 2022). Thus, the ability of mirabegron to inhibit adipocyte mesenchymal transition may be useful for the treatment of diseases other than metabolic syndrome with adipose dysfunction (Zhang et al. 2019; Marangoni et al. 2015, 2020; Zhu et al. 2022).

Our results raise questions about the mechanisms that regulate myofibroblasts in SC WAT in humans and how treatment with mirabegron antagonizes these pathways in vivo. TGFβ signaling is increased in adipose tissue with obesity (Marcelin et al. 2019; Roh et al. 2020; Jones et al. 2020), and may therefore be a significant regulator of myofibroblasts in vivo. TGFβ promotes adipocyte mesenchymal transition and regulates snail (Wang et al. 2013). A direct pathway for the reduction of myofibroblasts and*snail* expression in vivo is that mirabegron acts on adipocytes through the β3-AR to reduce myofibroblast transition, which is supported by our in vitro study. However, the mechanism could be more complicated than the inhibition of adipocyte mesenchymal transition since other cell types present in adipose tissue such as preadipocytes and endothelial cells can also undergo mesenchymal transition. Finally, this study suggests that inhibition of the classical pathway of epithelial mesenchymal transition is not involved in the reduction of adipose tissue myofibroblasts by mirabegron treatment. The potential role of the genes identified as having core enrichment (Table S5 through S8) in this process remains to be identified.

This study also revealed that CXCR2 was significantly lowered in SC WAT by mirabegron treatment, and this led us to identify NK cells as one of the major sites of CXCR2 expression in adipose tissue. NK cells have emerged as important immune cells in adipose tissue that promote macrophage inflammation and insulin resistance in response to adipocyte stress (Wensveen et al. 2015; Lee et al. 2016). Surprisingly, mirabegron treatment did not lower NK cell abundance but lowered the percentage of cells expressing CXCR2, indicating a change in NK cells towards a less inflammatory phenotype. Reducing CXCR2 on NK cells could thus explain the anti-inflammatory actions we previously observed in adipose tissue in response to mirabegron treatment, including the increase in anti-inflammatory macrophages (Finlin et al. 2020). Further studies will be necessary to determine the effect of reducing CXCR2 on NK cells in vivo.

Although this study has the advantage of studying adipose tissue from human subjects treated with a β3AR agonist, there are several limitations to the interpretation of the results. The SC WAT biopsies are from a study that did not contain a placebo control arm. The abundance of adipose tissue myofibroblasts is relatively small (approximately half that of adipose macrophages (Spencer et al. 2014), and the contribution of myofibroblasts to overall adipose tissue dysfunction is thus uncertain. This study identified a negative correlation between myofibroblast density and β cell function, but did not identify the mechanism for adipose tissue – β cell crosstalk. The results of this study also suggest that mirabegron treatment changed the NK phenotype, but further studies will be necessary to determine the effect of the reduction of CXCR2 expression on NK cell function.

Overall, this study uncovered previously unrecognized effects of the β3-AR agonist on adipose tissue in humans. This study also raises important questions about the specific roles that myofibroblasts play in adipose tissue dysfunction that occurs with obesity. Identifying the mechanisms leading to the reduction of SC WAT myofibroblasts and the change in NK cell phenotype in vivo are important future goals.

## Supplementary Information

Supplementary Material 1

Supplementary Material 2

Supplementary Material 3

Supplementary Material 4

Supplementary Material 5

Supplementary Material 6

Supplementary Material 7

Supplementary Material 8

Supplementary Material 9

Supplementary Material 10

Supplementary Material 11

Supplementary Material 12

Supplementary Material 13

Supplementary Material 14

Supplementary Material 15

Supplementary Material 16

Supplementary Material 17

Supplementary Material 18

Supplementary Material 19

Supplementary Material 20

## Data Availability

The data and resources from this study will be made available upon reasonable request.The RNA seq data are available at the gene expression omnibus (GSE272175).

## Abbreviations

ADHASCS: Adult-derived human adipocyte stem cells
AR: Adrenergic receptor
ASMA: Alpha smooth muscle actin
AT: Brown adipose tissue
BMI: Body mass index
cAMP: cyclic adenosine monophosphate
CXCR2: Chemokine receptor 2
GSEA: Gene set enrichment analysis
NK: Natural killer
SC WAT: Subcutaneous white adipose tissue
TGFβ: Transforming growth factor beta
WAT: White adipose tissue
