# Supplementary figures and images for: Mirabegron treatment reduces myofibroblasts and CXCR2 expression in adipose tissue in obesity
Source: Mol Med. 2025 Oct 14;31:313. doi: 10.1186/s10020-025-01368-2 (PMC12522344; doi:10.1186/s10020-025-01368-2)

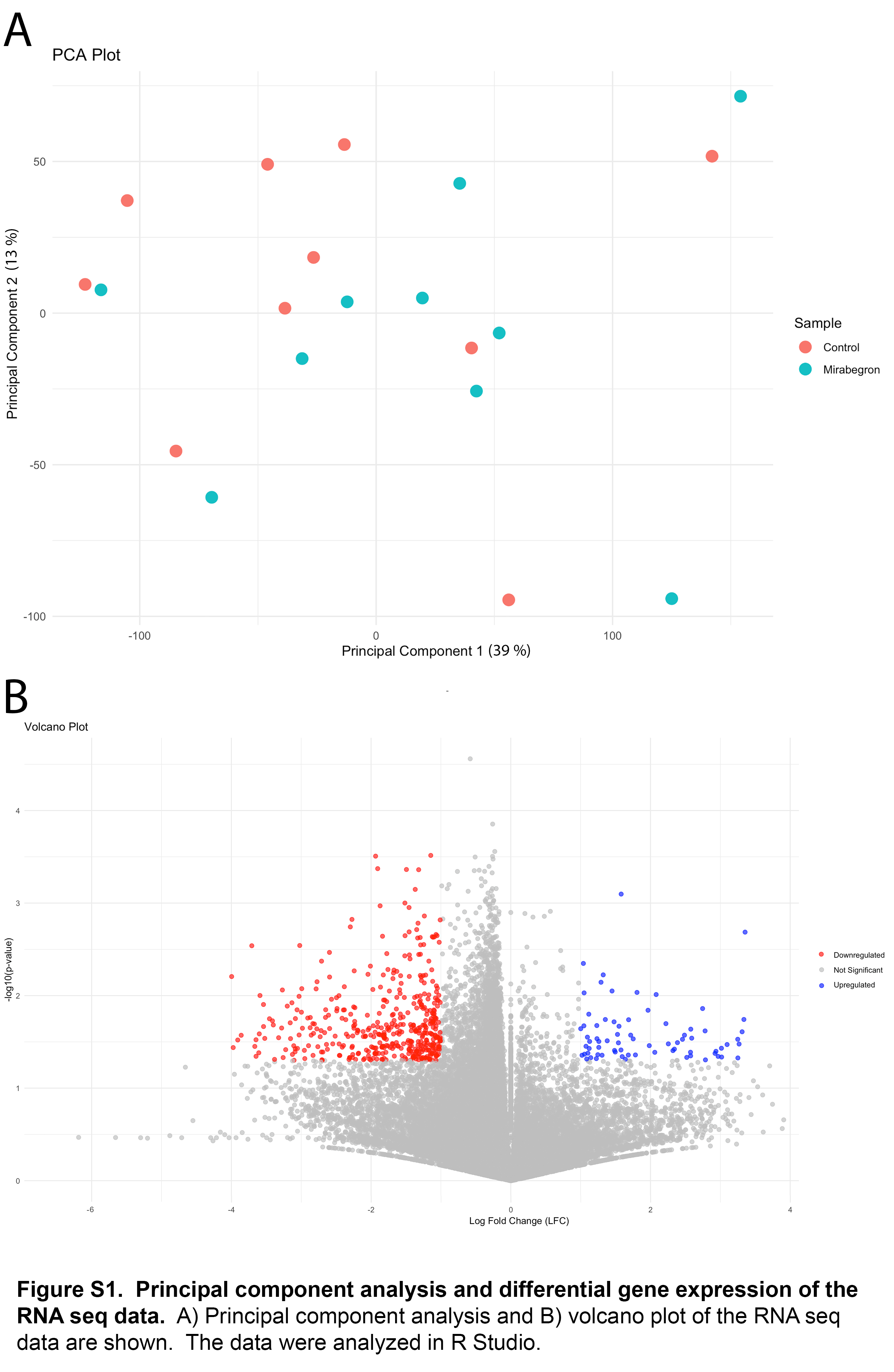

Supplement: Supplementary file 1 — Supplementary Material 1 [file 10020_2025_1368_MOESM1_ESM.tif]

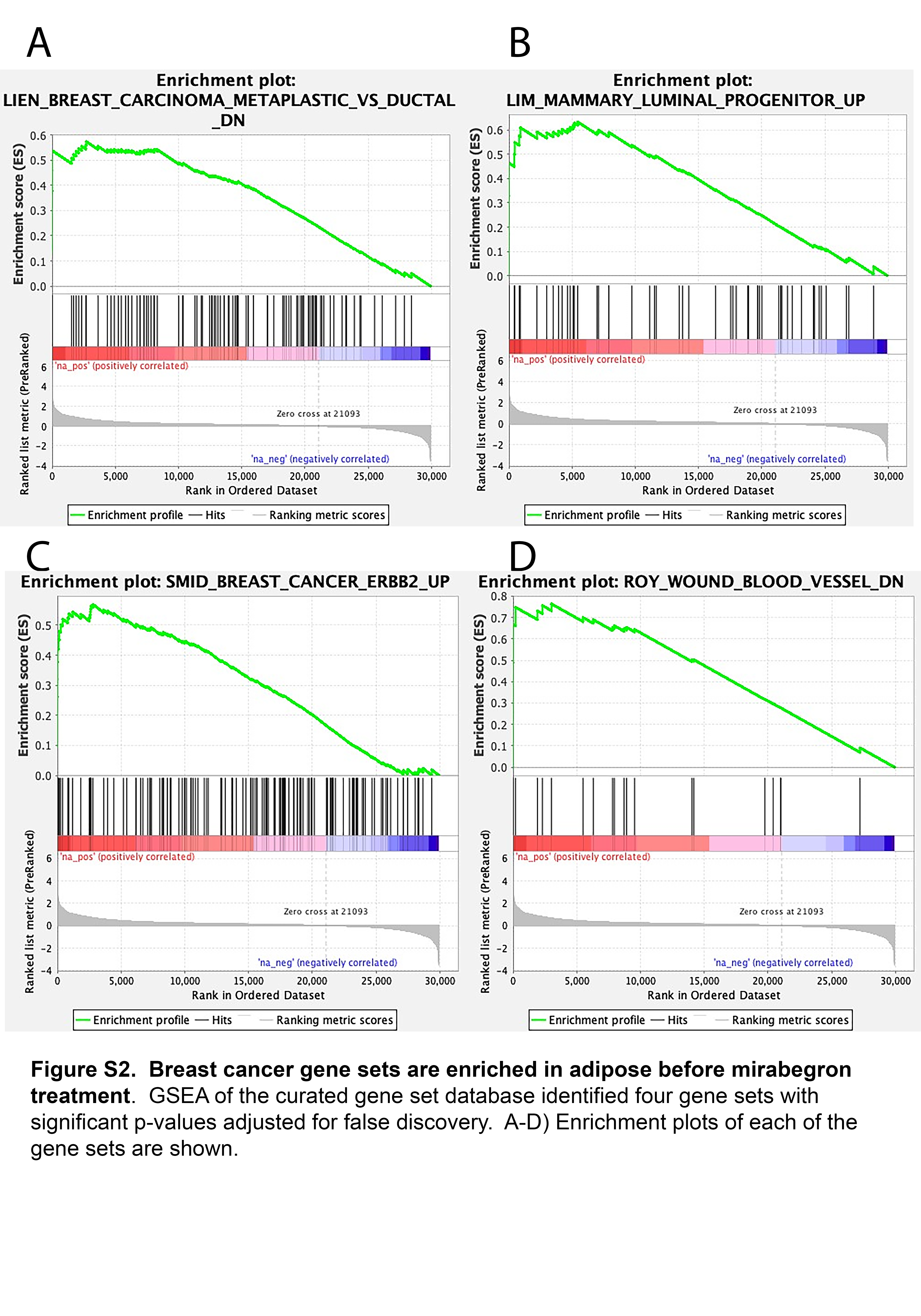

Supplement: Supplementary file 2 — Supplementary Material 2 [file 10020_2025_1368_MOESM2_ESM.tif]

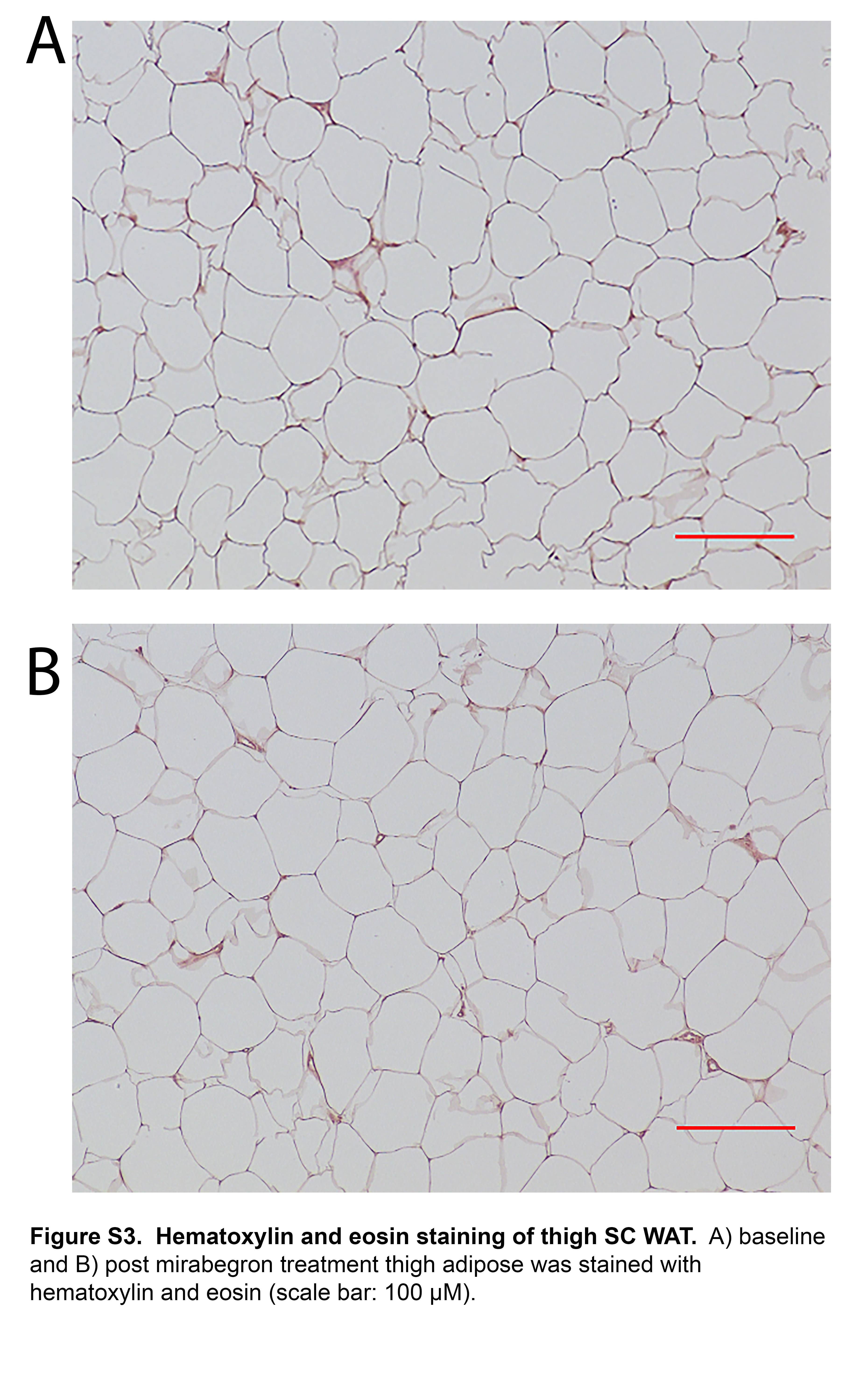

Supplement: Supplementary file 3 — Supplementary Material 3 [file 10020_2025_1368_MOESM3_ESM.tif]

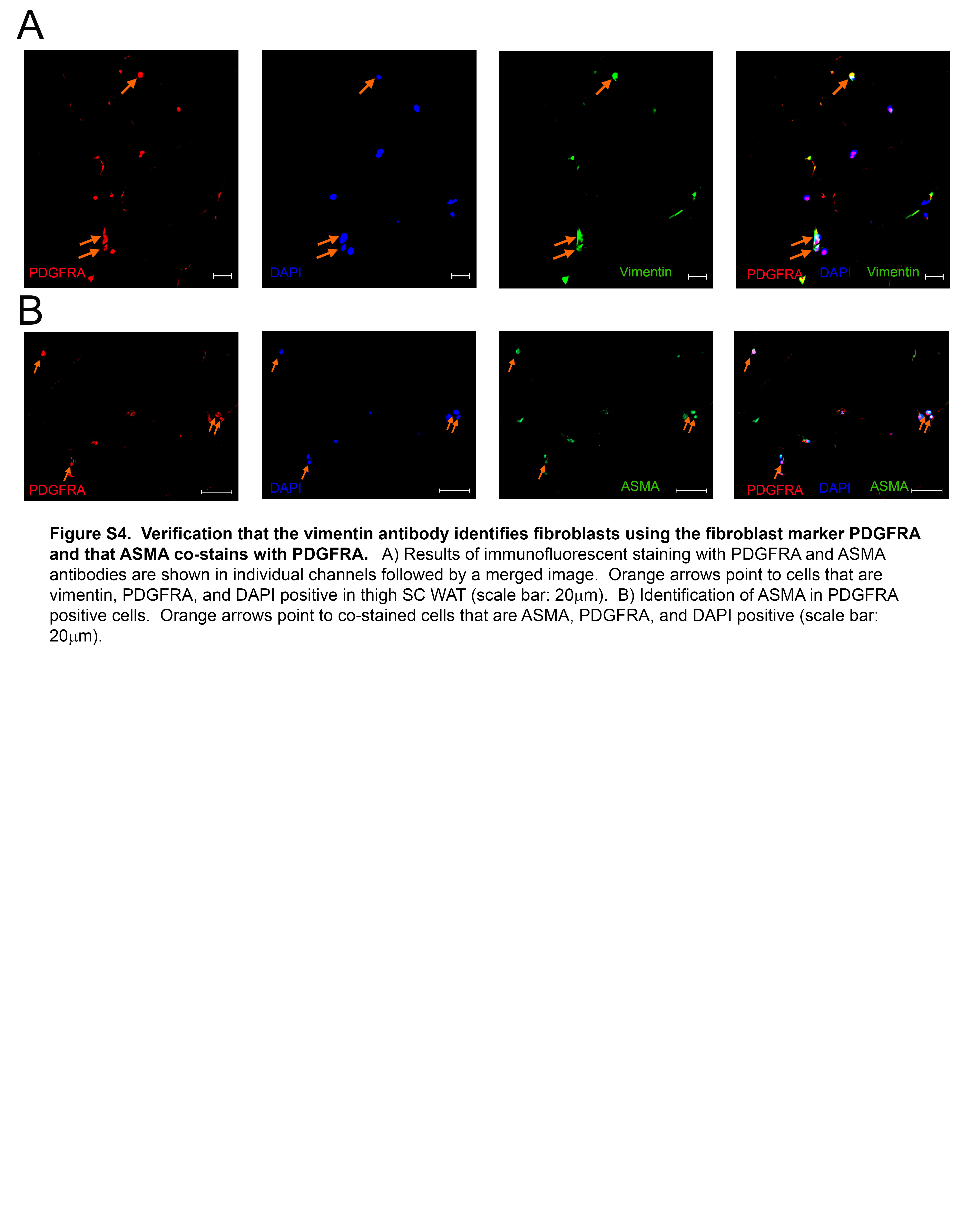

Supplement: Supplementary file 4 — Supplementary Material 4 [file 10020_2025_1368_MOESM4_ESM.tif]

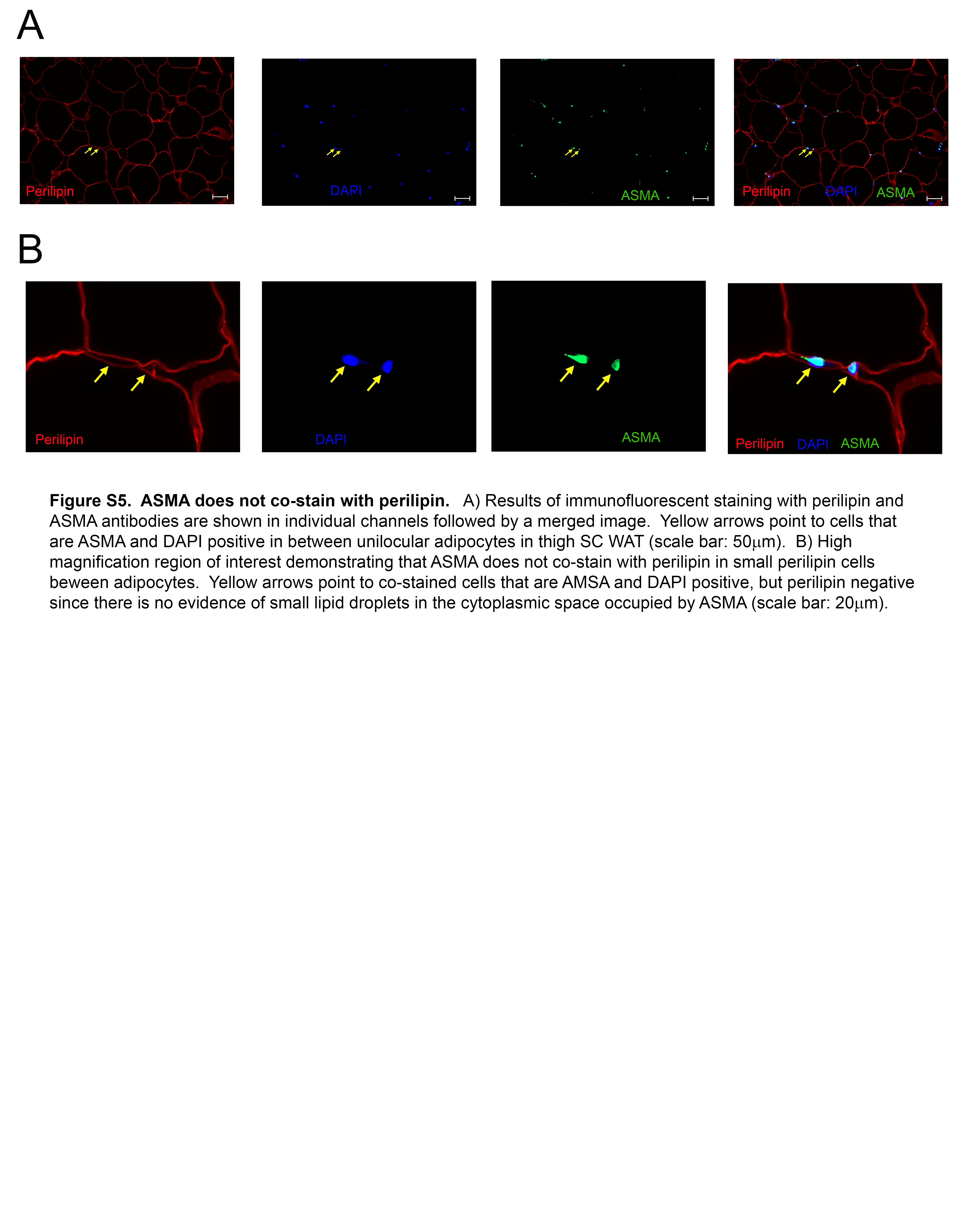

Supplement: Supplementary file 5 — Supplementary Material 5 [file 10020_2025_1368_MOESM5_ESM.tif]

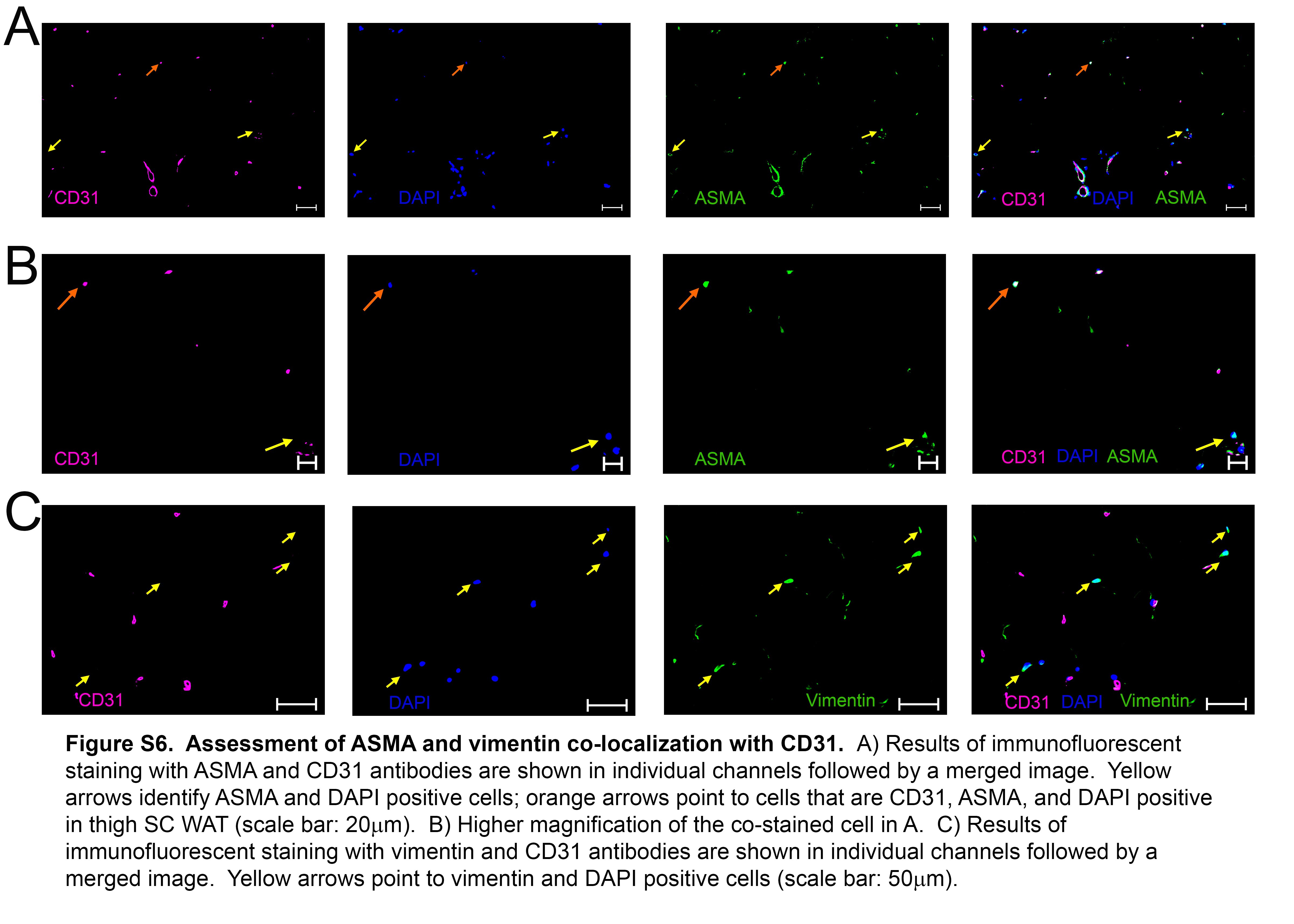

Supplement: Supplementary file 6 — Supplementary Material 6 [file 10020_2025_1368_MOESM6_ESM.tif]

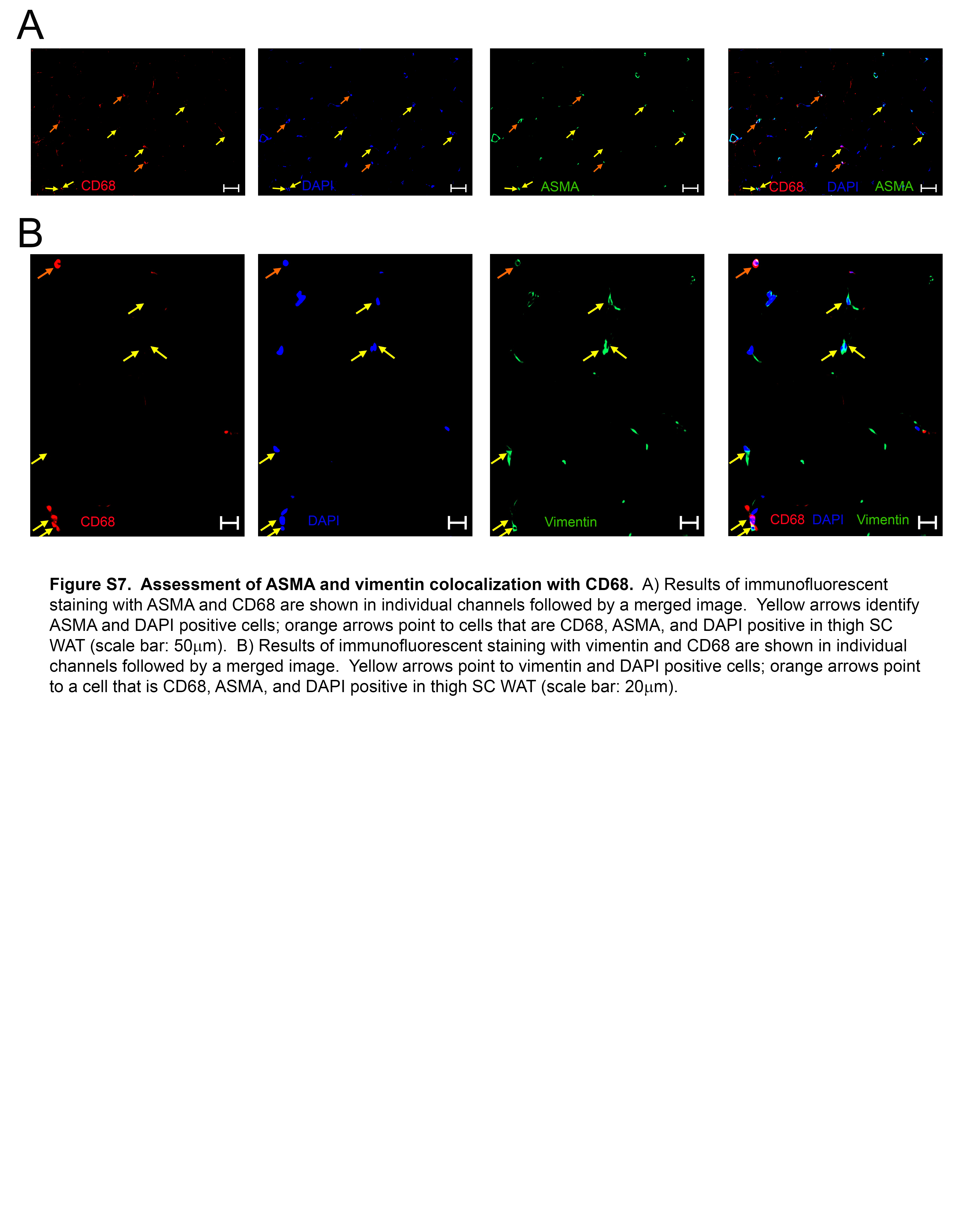

Supplement: Supplementary file 7 — Supplementary Material 7 [file 10020_2025_1368_MOESM7_ESM.tif]

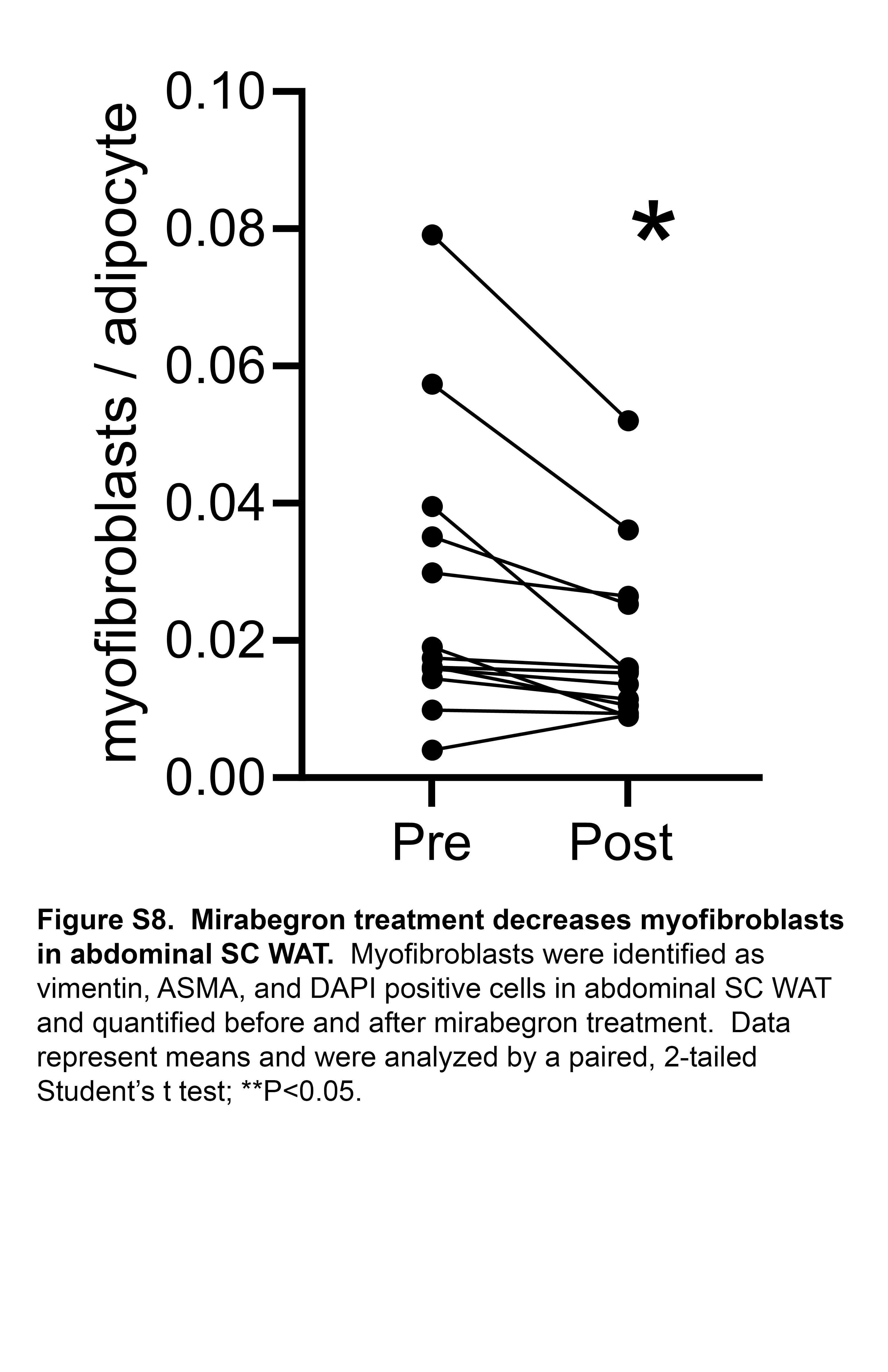

Supplement: Supplementary file 8 — Supplementary Material 8 [file 10020_2025_1368_MOESM8_ESM.tif]

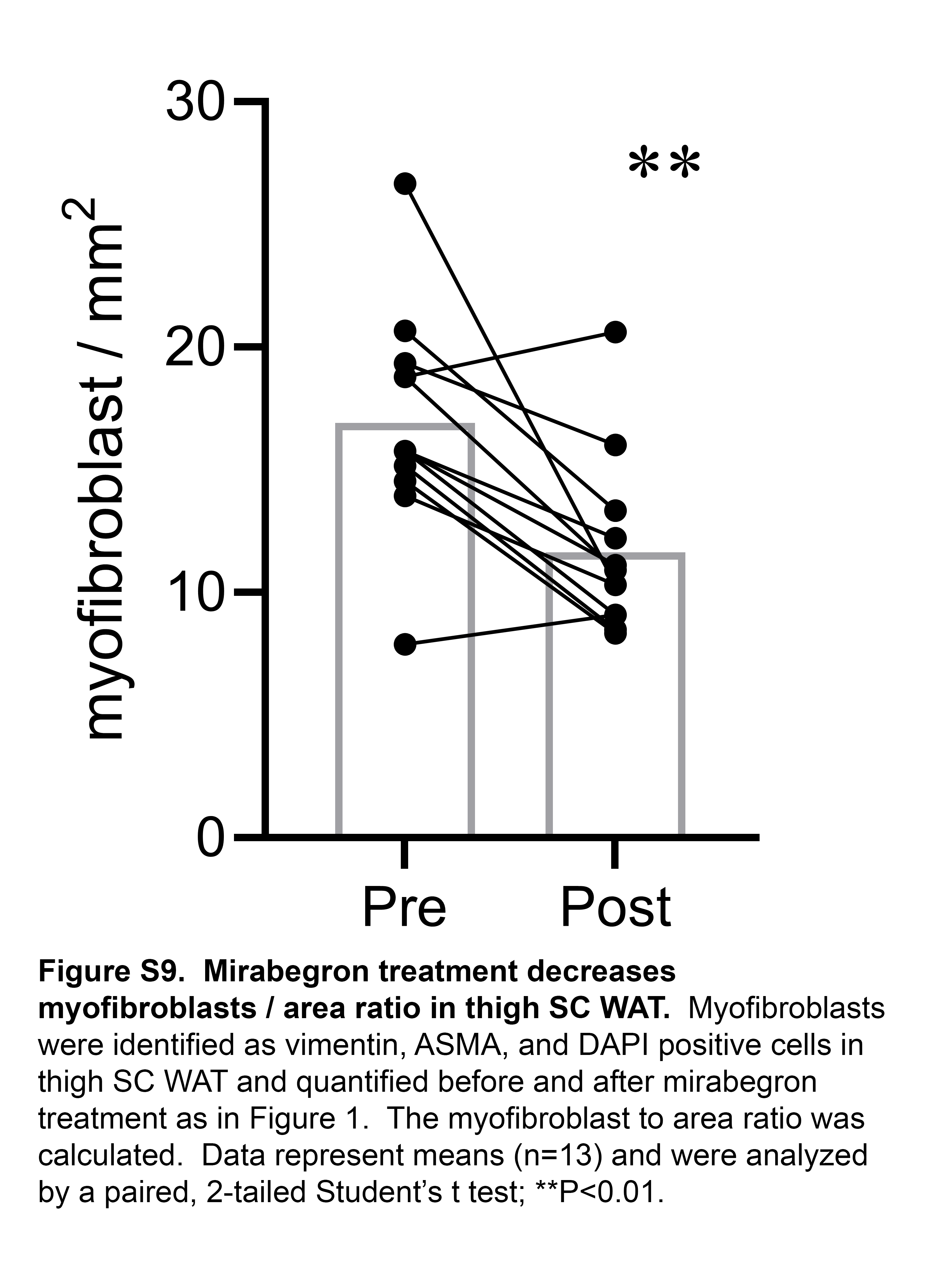

Supplement: Supplementary file 9 — Supplementary Material 9 [file 10020_2025_1368_MOESM9_ESM.tif]

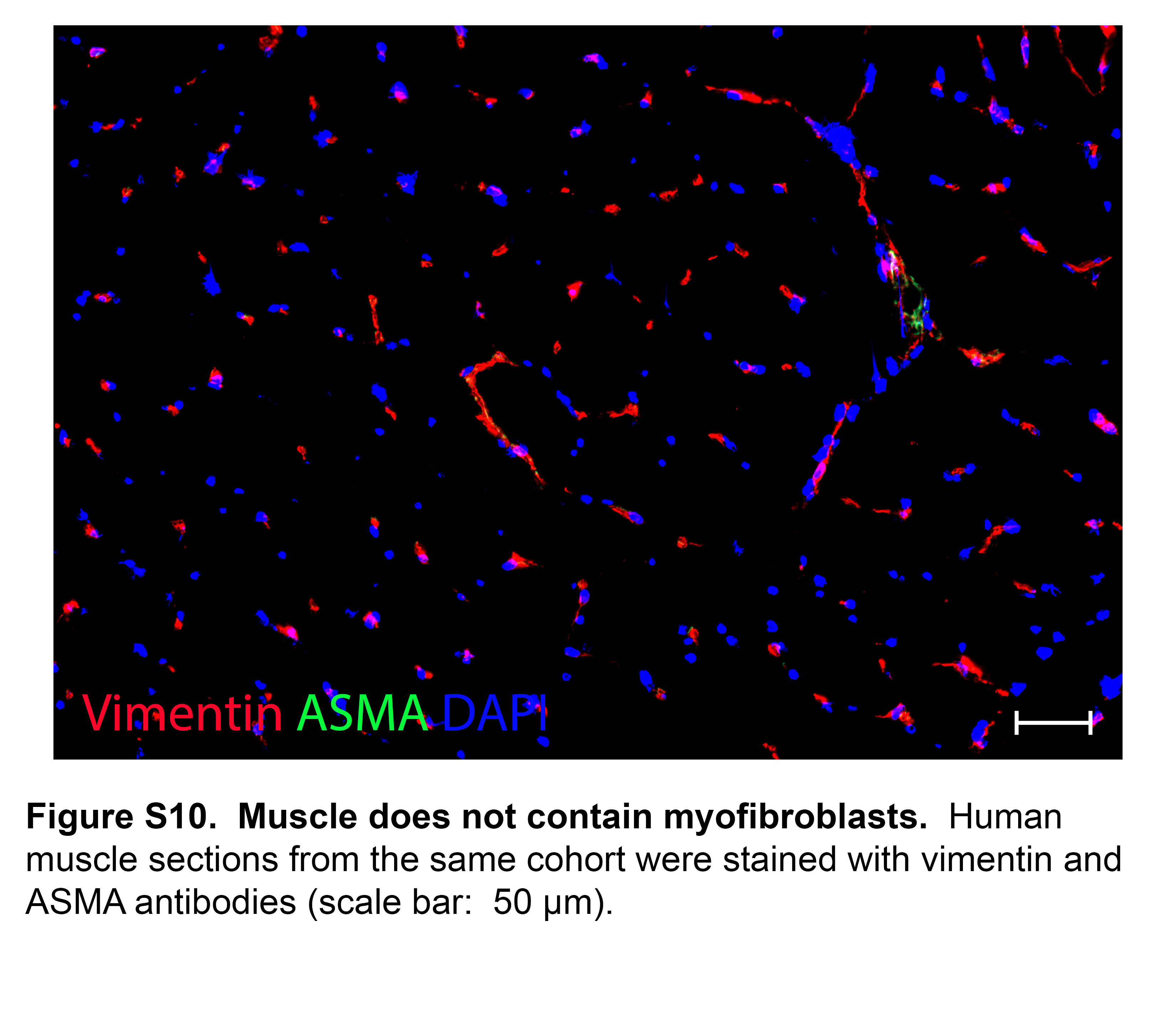

Supplement: Supplementary file 10 — Supplementary Material 10 [file 10020_2025_1368_MOESM10_ESM.tif]

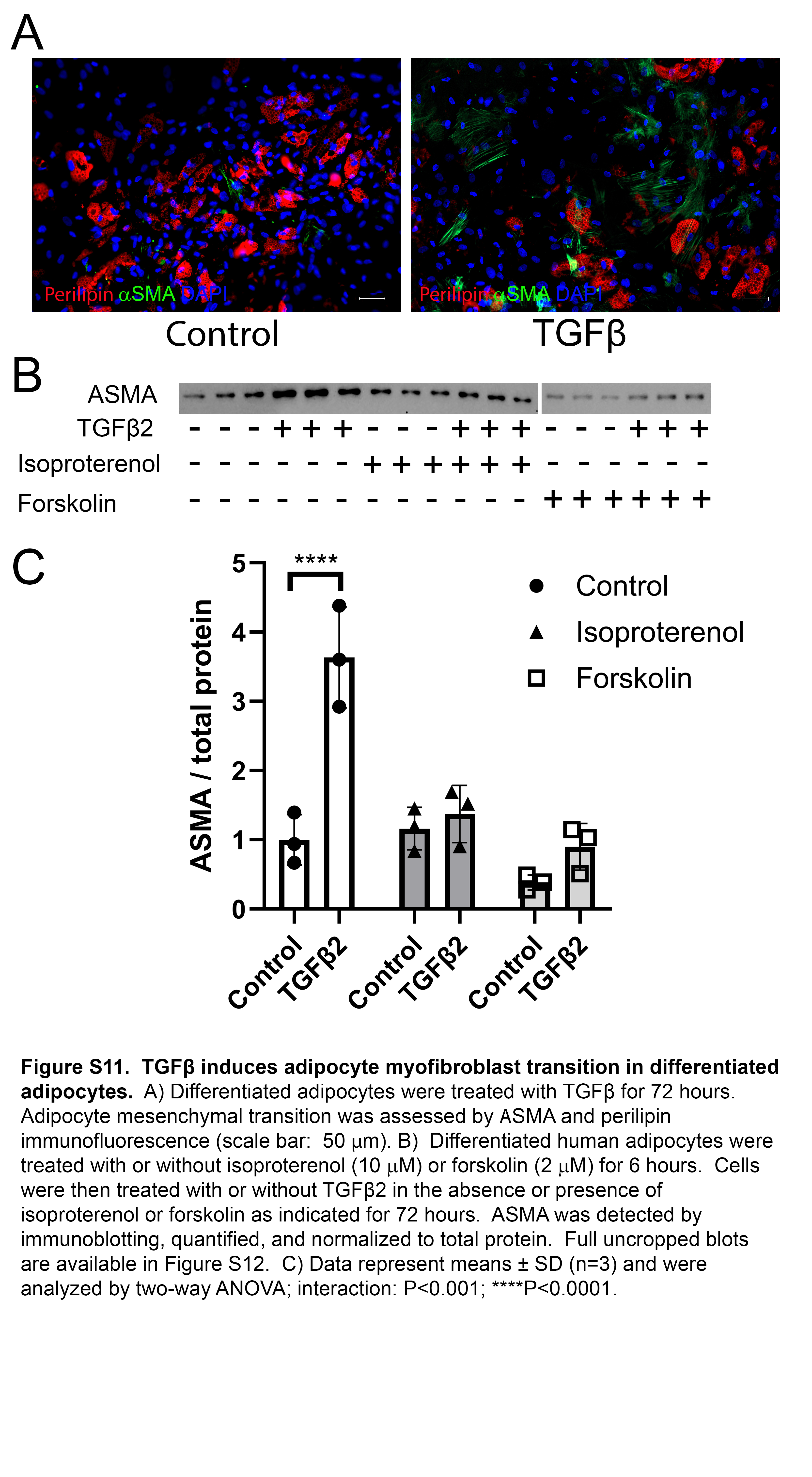

Supplement: Supplementary file 11 — Supplementary Material 11 [file 10020_2025_1368_MOESM11_ESM.tif]

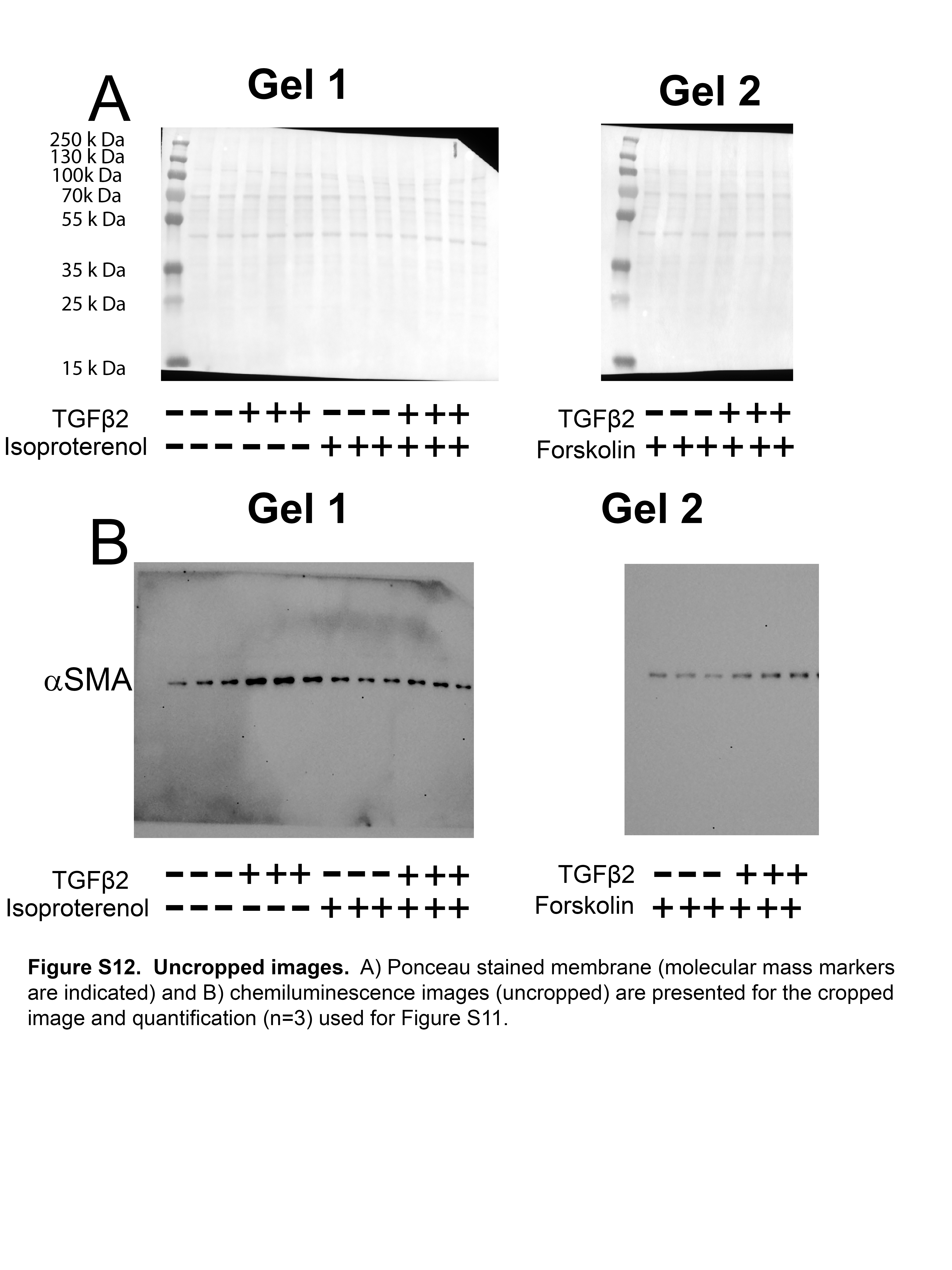

Supplement: Supplementary file 12 — Supplementary Material 12 [file 10020_2025_1368_MOESM12_ESM.tif]

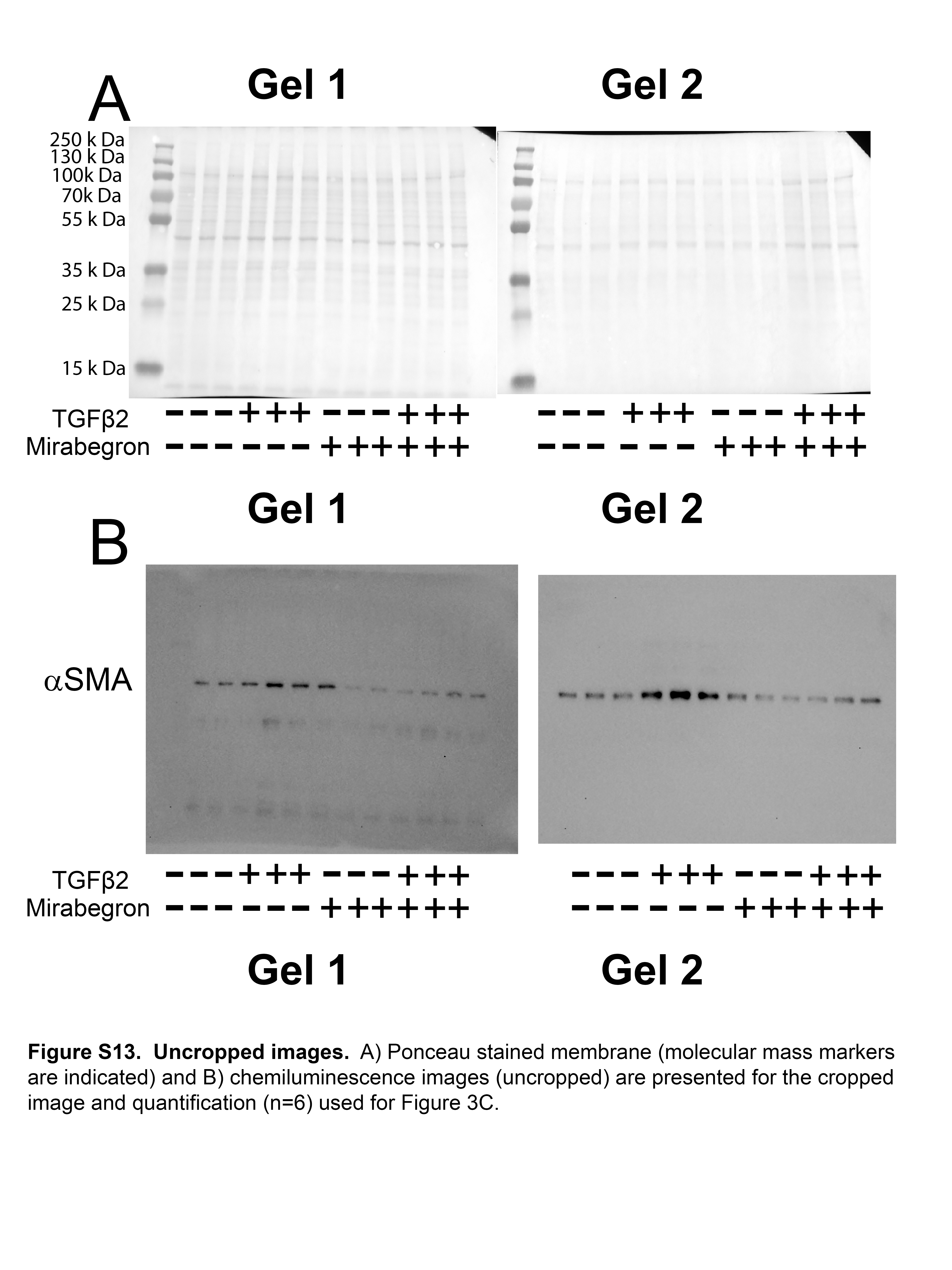

Supplement: Supplementary file 13 — Supplementary Material 13 [file 10020_2025_1368_MOESM13_ESM.tif]

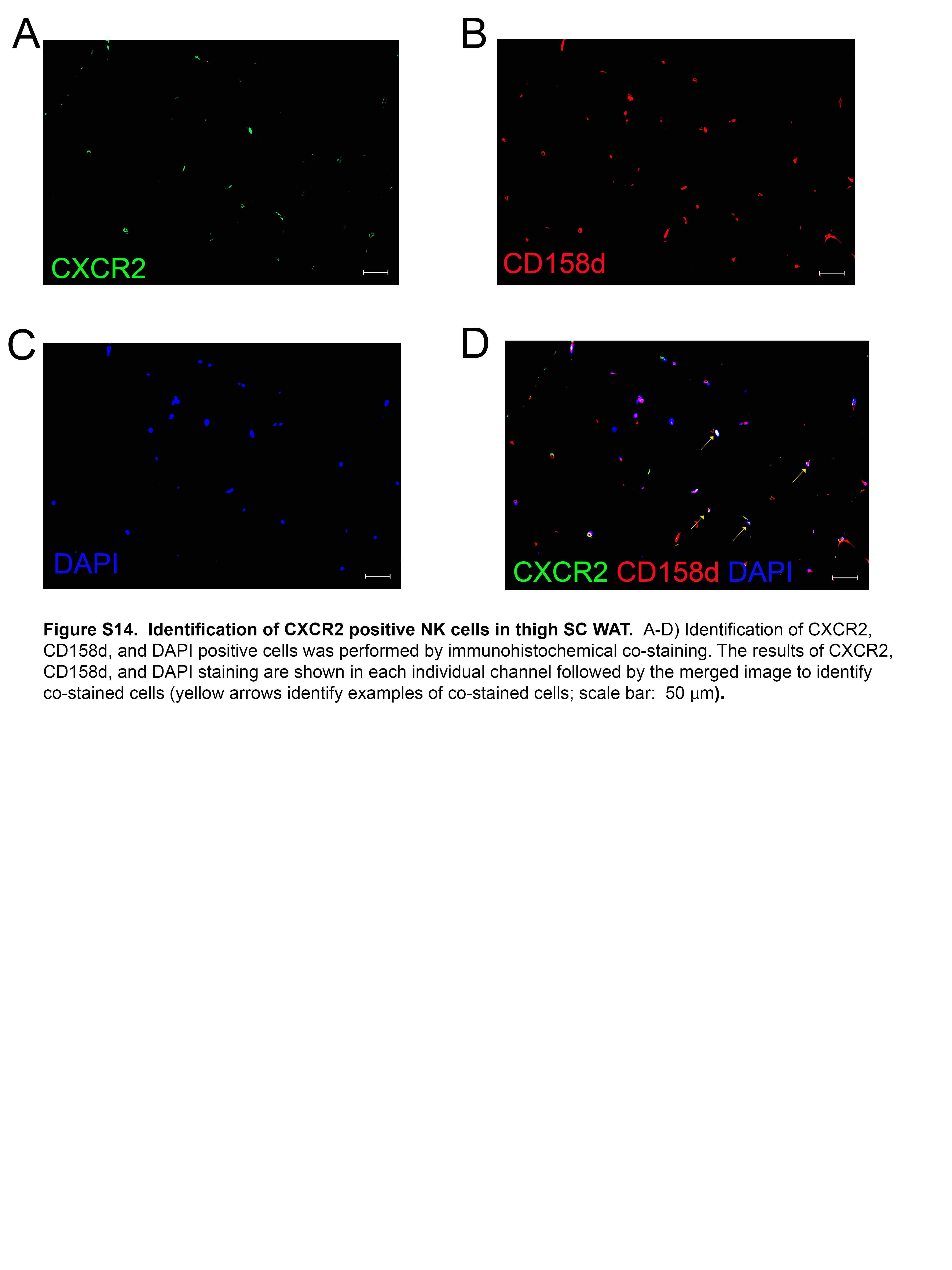

Supplement: Supplementary file 14 — Supplementary Material 14 [file 10020_2025_1368_MOESM14_ESM.tif]

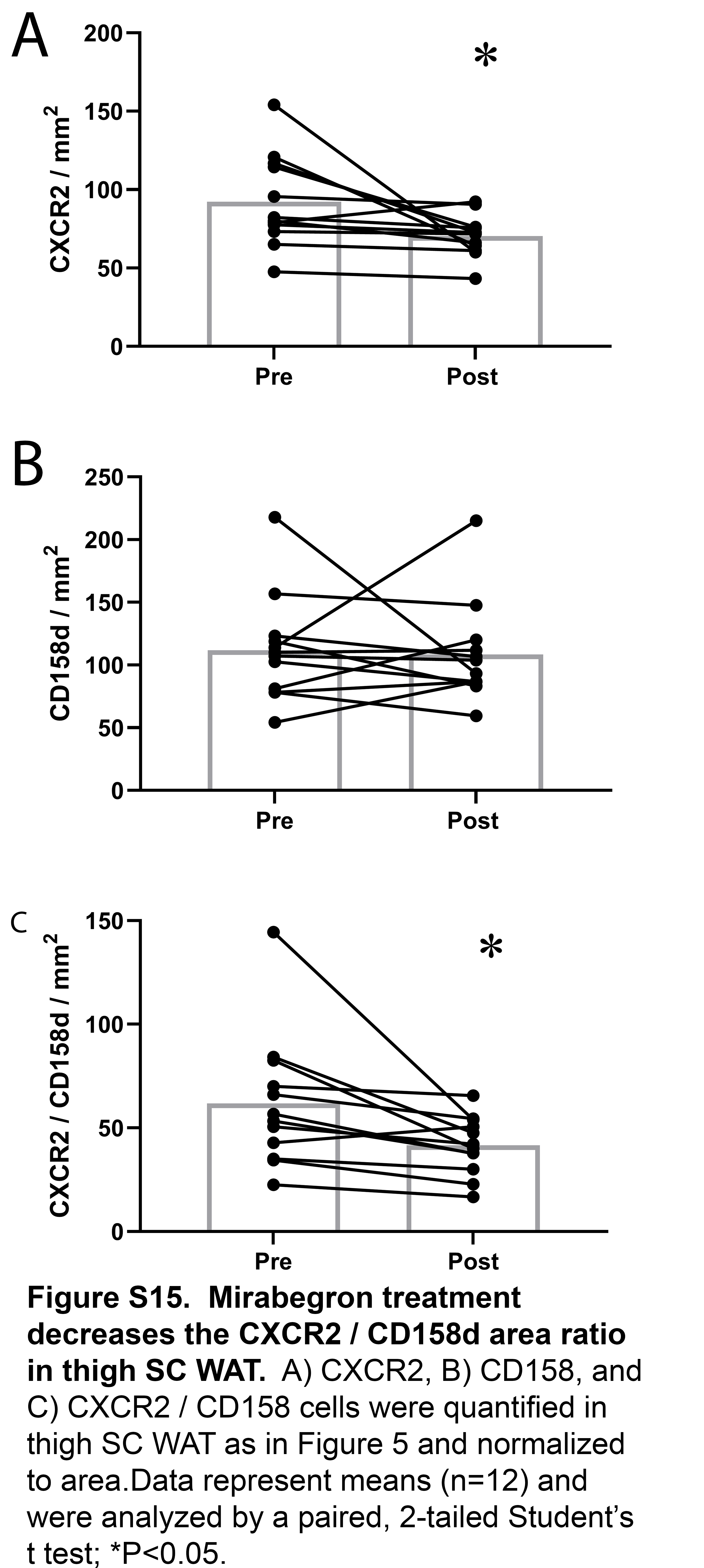

Supplement: Supplementary file 15 — Supplementary Material 15 [file 10020_2025_1368_MOESM15_ESM.tif]
