# Supplementary material for: Mirabegron treatment reduces myofibroblasts and CXCR2 expression in adipose tissue in obesity
Source: Mol Med. 2025 Oct 14;31:313. doi: 10.1186/s10020-025-01368-2 (PMC12522344; doi:10.1186/s10020-025-01368-2)
Supplement: Supplementary file 16 — Supplementary Material 16 [file 10020_2025_1368_MOESM16_ESM.docx]

Table S1. Primer sequences.

| Gene  symbol | Forward | Reverse |
| --- | --- | --- |
| ACTB | GAGCACAGAGCCTCGCCTTT | CGCGGCGATATCATCATCCAT |
| PPIA | CCCACCGTGTTCTTCGACAT | GCTGTCTTTGGGACCTTGTCT |
| PPIB | AAGTCACCGTCAAGGTGTATTTT | TGCTGTTTTTGTAGCCAAATCCT |
| TBP | CCCGAAACGCCGAATATAATCC | AATCAGTGCCGTGGTTCGTG |
| TUBB | ACCAACCTACGGGGATCTGAA | TTGACTGCCAACTTGCGGA |
| UBC9 | CTGGAAGATGGTCGTACCCTG | GGTCTTGCCAGTGAGTGTCT |
| CXCR1 | CTGACCCAGAAGCGTCACTTG | CCAGGACCTCATAGCAAACTG |
| CXCR2 | CCTGTCTTACTTTTCCGAAGGAC | TTGCTGTATTGTTGCCCATGT |
| SNAI1 | TCGGAAGCCTAACTACAGCGA | AGATGAGCATTGGCAGCGAG |
